# Multivariate Longitudinal Modeling of Macular Ganglion Cell Complex: Spatiotemporal Correlations and Patterns of Longitudinal Change

**DOI:** 10.1016/j.xops.2022.100187

**Published:** 2022-06-16

**Authors:** Vahid Mohammadzadeh, Erica Su, Lynn Shi, Anne L. Coleman, Simon K. Law, Joseph Caprioli, Robert E. Weiss, Kouros Nouri-Mahdavi

**Affiliations:** 1 Glaucoma Division, Stein Eye Institute, David Geffen School of Medicine, University of California Los Angeles, Los Angeles, California; 2 Department of Biostatistics, Fielding School of Public Health, University of California Los Angeles, Los Angeles, California

**Keywords:** Bayesian, Ganglion cell complex, Hierarchical, Longitudinal, Macular OCT, GCC, ganglion cell complex, PC, principal component, RNFL, retinal nerve fiber layer, SD, standard deviation, VF, visual field

## Abstract

**Purpose:**

To investigate spatiotemporal correlations among ganglion cell complex (GCC) superpixel thickness measurements and explore underlying patterns of longitudinal change across the macular region.

**Design:**

Longitudinal cohort study.

**Subjects:**

One hundred eleven eyes from 111 subjects from the Advanced Glaucoma Progression Study with ≥ 4 visits and ≥ 2 years of follow-up.

**Methods:**

We further developed our proposed Bayesian hierarchical model for studying longitudinal GCC thickness changes across macular superpixels in a cohort of glaucoma patients. Global priors were introduced for macular superpixel parameters to combine data across superpixels and better estimate population slopes and intercepts.

**Main Outcome Measures:**

Bayesian residual analysis to inspect cross-superpixel correlations for subject random effects and residuals. Principal component analysis (PCA) to explore underlying patterns of longitudinal macular change.

**Results:**

Average (standard deviation [SD]) follow-up and baseline 10-2 visual field mean deviation were 3.6 (0.4) years and −8.9 (5.9) dB, respectively. Superpixel-level random effects and residuals had the greatest correlations with nearest neighbors; correlations were higher in the superior than in the inferior region and strongest among random intercepts, followed by random slopes, residuals, and residual SDs. PCA of random intercepts showed a first large principal component (PC) across superpixels that approximated a global intercept, a second PC that contrasted the superior and inferior macula, and a third PC, contrasting inner and nasal superpixels with temporal and peripheral superpixels. PCs for slopes, residual SDs, and residuals were remarkably similar to those of random intercepts.

**Conclusions:**

Introduction of cross-superpixel random intercepts and slopes is expected to improve estimation of population and subject parameters. Further model enhancement may be possible by including cross-superpixel random effects and correlations to address spatiotemporal relationships in longitudinal data sets.

Detection of glaucoma progression is crucial for prevention of irreversible visual loss.^1^^,^^2^ To this aim, it is essential to identify eyes with rapid or central glaucoma progression.^3^ Macular OCT imaging is now considered the standard method for assessing the health of the central retinal ganglion cells.^4^, ^5^, ^6^, ^7^, ^8^, ^9^ There is mounting evidence that macular OCT imaging can provide additional information complementary to that provided by retinal nerve fiber layer (RNFL) thickness measurements for detection of glaucoma progression, especially after the early stages of glaucoma.^2^^,^^3^^,^^5^^,^^10^^,^^11^

Our research group has shown that ganglion cell complex (GCC), which is the sum of macular RNFL, ganglion cell layer, and inner plexiform layer, could be the preferred outcome measure for establishing glaucoma progression in the macular region.^12^^,^^13^ We recently proposed and implemented a random intercept and random slope Bayesian hierarchical model for estimating population average and individual rates of change at the level of 3° × 3° macular superpixels in a cohort of patients with central damage or moderate-to-advanced glaucoma. Our model also included a subject-specific random variance for superpixel residuals.^13^, ^14^, ^15^, ^16^, ^17^, ^18^, ^19^ In this model, information from the entire cohort is used to efficiently estimate individual intercepts and slopes for each superpixel. Compared with simple linear regression of thickness measurements against time in a single superpixel for a single subject, the hierarchical model more efficiently estimates individual slopes and provides population estimates.^14^, ^15^, ^16^, ^17^, ^18^

Our initial random intercept and random slope model was fit to all subjects’ longitudinal data from a single superpixel and did not attempt to model correlations of population or individual parameters across superpixels.^13^ Although the cross-sectional correlation of the 10-2 visual field (VF) test locations has been previously explored,^20^ there is no information in the published literature on cross-sectional or longitudinal correlations among thickness measurements of superpixels across the macula. Constructing an appropriate longitudinal model for macular thickness that incorporates spatial and temporal relationships holds the possibility of improved estimation of subject-level slopes over the single superpixel hierarchical model and potentially allows us to make accurate global statements about the probability of glaucoma progression across the macula.

At issue with constructing a spatial–temporal model is what kind of spatial structure to incorporate.^12^^,^^21^^,^^22^ Thus, we further developed and fit a hierarchical random-effects model that allows for spatial relationships to be discovered without forcing a preconceived notion of which superpixels might be most similar. We use this joint model to explore the spatial correlations of the 3 random effects and the residuals.

The aim of this study is (a) to develop a single Bayesian multivariate hierarchical model encompassing data from all superpixels over time with a hierarchical prior for the individual superpixel parameters and (b) to estimate correlations across superpixels of the 3 subject-specific random effects (intercept, slope, and residual superpixel variance) in our initial model and the subject-visit residuals in the proposed hierarchical model. Such information will allow us to incorporate appropriate spatial–temporal correlations into our Bayesian hierarchical model.

## Methods

### Study Sample

We enrolled 111 eyes from 111 subjects from the Advanced Glaucoma Progression Study, an ongoing longitudinal study at the University of California, Los Angeles. Eligible eyes were required to have a minimum of 4 macular OCT images, ≥ 2 years of follow-up, and no other ocular pathology at baseline and during follow-up. We analyzed observations up to 4.2 years after baseline in this study. We omitted all data from visits less than 0.2 years after a previous visit.^13^

The study adhered to the tenets of the Declaration of Helsinki, was approved by University of California, Los Angeles’s Human Research Protection Program, and conformed to the Health Insurance Portability and Accountability Act policies. All subjects provided written informed consent at the time of enrollment in the study.

Inclusion criteria were as follows: (a) clinical diagnosis of primary open-angle glaucoma, pseudoexfoliative glaucoma, pigmentary glaucoma, or primary angle-closure glaucoma; (b) evidence of either central damage on the 24-2 VF, defined as 2 or more points within the central 10° with *P* < 0.05 on the pattern deviation plot or VF mean deviation worse than −6 dB. Exclusion criteria consisted of baseline age less than 40 years or greater than 80 years, best-corrected visual acuity < 20 of 50, refractive error exceeding 8 diopters (D) of the sphere or 3 D of the cylinder, or significant retinal or neurological disease affecting OCT measurements.

### Imaging Procedures

The posterior pole algorithm of Spectralis spectral domain OCT (Heidelberg Engineering) was used to image the macular region and estimate GCC thickness. The posterior pole algorithm volume scan spans a 30° × 25° region and consists of 61 B-scans spaced approximately 120 μm apart. Each B-scan was repeated 9 to 11 times to reduce speckle noise. Segmentation was performed with the Glaucoma Module Premium Edition, the built-in software of the Spectralis OCT. After manual correction of incorrect segmentations, 8 × 8 arrays of individual layer thickness measurements at 3° × 3° superpixels from the central 24° × 24° of the measurement cube were exported. The GCC thickness was calculated by adding the macular RNFL, ganglion cell layer, and inner plexiform layer. We included a 7 × 7 subarray of superpixels in this study, omitting the most inferior row and the nasal-most column due to high numbers of reported zeros and observed variability in prior studies.^12^^,^^23^

### A Multivariate Hierarchical Longitudinal Model

Our data cleaning methods have been described previously, and we provide details in the web appendix.^13^ We inspected profile plots and empirical summary plots of outcomes for all subjects and all superpixels.^15^ We removed zero values as erroneous, and we identified and removed outliers that caused large increases/decreases between consecutive measurements; details of the outlier removal algorithm are given in the web appendix section titled “Outlier Removal Algorithm for 49 Superpixels.” Subject profiles in each superpixel that had 2 or more identified outliers were completely removed from the analysis.

We previously developed a Bayesian normal hierarchical random-effects model of longitudinal GCC measurements for a cohort of *n* subjects indexed by *i* = 1, …, *n*.^13^ Our multivariate proposed model has 7 parameters for each superpixel: a population intercept, population slope, subject-specific random intercept variance, subject-specific random slope variance and random intercept/slope correlation, and 2 population parameters, a mean and a variance, to model the subject-specific random residual variances. We explore a hierarchical random-effects model that allows information from all superpixels to help estimate these 7 parameters in each individual superpixel. For each subject in each superpixel, the model has 3 subject-level random effects: intercept, slope, and residual variance. At each subject visit, there are subject residuals across superpixels. These random effects and residuals likely have spatial correlations.

Observations *y*_*ijk*_ for subject *i* at time *t*_*ij*_ are GCC thickness (μm) in superpixel *k* from among *K* superpixels. This model has subject-specific random intercepts β_0ik_ and random slopes β_1ik_ and subject-specific residual variances $\sigma_{ik}^{2}$ in superpixel *k*. There are 7 interpretable population parameters in the *k*^th^ superpixel: (i) the population intercept *α*_0*k*_, (ii) the random intercept standard deviation (SD) $D_{00k}^{1/2}$, (iii) population slope α_1k_, (iv) the random slope SD $D_{11k}^{1/2}$, (v) the correlation $\rho_{k}=D_{01k}D_{00k}^{-1/2}D_{11k}^{-1/2}$ between the random intercepts and slopes, where *D*_01*k*_ is the covariance of the random intercepts and slopes in superpixel *k*, and (vi and vii) the population mean $\sigma_{mk}$ and SD $\sigma_{sk}$ of the subject-specific residual SDs $\sigma_{ik}$. For superpixel *k* and subject *i*, the model is linear in time,

$${\text{y}}_{\text{ijk}}={\text{α}}_{0\text{k}}+{\text{α}}_{1\text{k}}{\text{t}}_{\text{ij}}+{\text{β}}_{0\text{ik}}+{\text{β}}_{1\text{ik}}{\text{t}}_{\text{ij}}+\varepsilon_{\text{ijk}}$$

where $\varepsilon_{\text{ijk}}\;∼\;N\left(0,\;\sigma_{ik}^{2}\right)$, and each subject follows their own subject-specific line.

We extended our single superpixel hierarchical model to one that included superpixel-level random effects for the 7 parameters. Prior specification and computation used a transformation (transformed parameters) of the 7 interpretable parameters to make normal priors more appropriate; an advantage of the Bayesian paradigm is that transformation back to the interpretable parameters is straightforward, which is discussed in the web appendix. Each transformed parameter has an unknown global mean and global SD, and we back-transform to the 7 interpretable parameters to report inferences.

Initially for the 7 transformed parameters, we set a 7-variate normal hierarchical prior with unknown mean *μ* and variance–covariance matrix Σ with proper but vague priors for *μ* and Σ. The Markov chain Monte Carlo algorithm implemented in Just Another Gibbs Sampler (JAGS) for this model had poor convergence. Inspection of the posterior correlations of Σ suggested that 3 of the 7 transformed parameters α_0k_, $\text{log}\left(D_{00k}\right)$, and a transformed variance parameter $\text{log}\left(D_{11.0k}\right)$, the remaining random slope variance after accounting for the correlation with random intercepts and a function of the random effect variance/covariance parameters *D*_00*k*_, *D*_11*k*_, and *D*_01*k*_, were highly correlated; on the other hand, each of the other 4 could be treated as a priori independent of each other and of the 3 correlated parameters. We proceeded to fit this reduced model, and we report these results. Priors for the mean, variances, and correlations of the parameters were proper but vague. Details of the prior, parameter transformations, and full model specification are given in the web appendix. The Bayesian hierarchical model was fit with JAGS using the *R2Jags* package in R, and we also estimated correlations and covariances of random effects and residuals in R.^24^, ^25^, ^26^

We fit the model using Markov Chain Monte Carlo with 3 chains of length 150 000 after burn-in of 50 000 and a thinning of 50, producing a posterior sample of size 9000 with satisfactory convergence. Using our prior published model, we refit individual data from the 49 superpixels to compare to the results from multiple superpixel analysis.^13^

### Exploration of Correlations Among Superpixels

Random-effects models and Bayesian methods allow for residual analysis, which is an important part of model checking and model development and elaboration.^17^^,^^27^, ^28^, ^29^ Within a Bayesian framework, we are able to estimate correlations between pairs of superpixels of subject-specific random intercepts, random slopes, log residual variances, and subject-visit residuals producing 4 covariance matrices. Given the 7 × 7 = 49 macular superpixels included in the current analysis, these correlation (covariance) matrices form a 49 × 49 correlation (covariance) matrix. We plotted the correlations in a correlogram, constructed as 49 7 × 7 heat maps of the correlations between each superpixel in turn with all other superpixels.^13^ Details of the correlation (covariance) matrix computations are presented in the web appendix.

### Principal Component Analyses

We then took the principal component (PC) decomposition, also known as an eigenvector/eigenvalue decomposition, of the 4 covariance matrices described here and inspected the eigenvectors corresponding to the largest PCs for interpretability. We plotted the eigenvectors in a set of 7 × 7 labeled heat maps. We drew scree plots for all 4 PC decompositions; scree plots display the cumulative variance explained by the PCs as a fraction of the total variance (sum of the 49 eigenvalues) against the PC’s rank. Inspection of the eigenvectors, eigenvalues, heat maps of eigenvectors, and scree plots indicates how to add additional components to our model.

## Results

From a total of 39 935 GCC superpixel measurements, we removed 7 zeros, 173 single outliers, and 130 observations from 18 outlying profiles for a total of 310 observations. We then analyzed 39 625 superpixel measurements in 49 superpixels from 815 visits of 111 subjects in a single model. The median (range) follow-up time was 3.62 (1.94–4.20) years with an average of 7.3 (range = 4–10) visits per subject. Table 1 provides a summary of the demographic and clinical characteristics of the cohort. The average (SD) age and baseline 10-2 VF mean deviation were 66.9 (8.5) years and −8.9 (5.9) dB.

**Table 1:** The Demographic and Clinical Characteristics of the Study Sample

Age (years)	
Mean (SD)	66.9 (8.5)
Range	39.7–81.2
Gender (%)	
Female	70 (63.1%)
Male	40 (36.0%)
Not reported	1 (0.9%)
Ethnicity (%)	
Caucasian	59 (53.2%)
Asian	24 (21.6%)
African American	15 (13.5%)
Hispanic	13 (11.7%)
Baseline 10-2 MD (dB)	
Median (IQR)	−7.6 (−12.0 to −4.1)
Mean (SD)	−8.9 (5.9)
Range	−25.1 to −0.4
Baseline 24-2 MD (dB)	
Median (IQR)	−6.7 (−12.3 to −4.3)
Mean (SD)	−8.7 (6.1)
Range	−26.4 to −0.3
Follow-up (years)	
Mean (SD)	3.59 (0.44)
Range	1.94–4.20
Visits per subject	
Mean (SD)	7.3 (1.1)
Range	4–10
Signal strength	
Mean (SD)	27.8 (3.1)
Range	21–36
Baseline GCC (μm)	
Mean (SD)	73.1 (20.1)
Range	37–154

GCC = ganglion cell complex; IQR = interquartile range; MD = mean deviation; SD = standard deviation.

**Figure 1:**
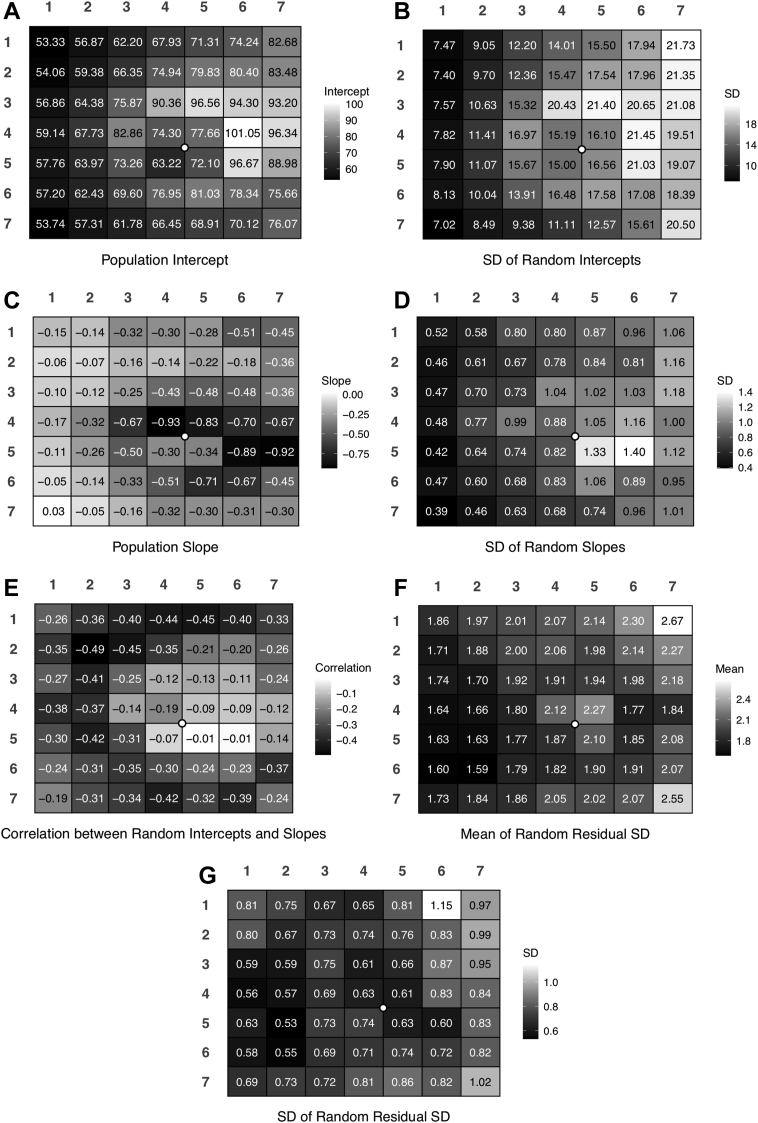
Heat maps of posterior mean (**A**) population intercepts, (**B**) SD of random intercepts, (**C**) population slopes, (**D**) SD of random slopes, (**E**) correlation between random intercepts and slopes, (**F**) mean of the random residual SD, and (**G**) SD of the random residual SD. The white circle indicates the fovea for visual orientation. SD = standard deviation.

Table 2 provides posterior summaries (mean, SD, 95% credible interval) of the global mean and SD parameters across superpixels of the 7 interpretable parameters plus correlations between 3 parameters, and Table S1 lists posterior summaries (mean, SD, 95% credible interval) of the transformed parameter global means, SDs, and correlations. Posterior means (SDs) for the most important global parameters were as follows. Superpixel population intercepts had global mean thickness of 73.1 (1.9) μm and global SD of 13.3 (1.4) μm across superpixels. Superpixel population slopes had global mean of −0.36 (0.04) microns per year and global SD of 0.27 (0.03) across superpixels. Figure 1 provides heat maps of the posterior means of the superpixel-level population intercepts, slopes, random intercept and slope SDs, random intercept and slope correlations, and mean and SD of the random residual SDs. Fig S1 plots posterior means of the population parameters from the current model against estimates derived from our original model based on separate analyses of data from each superpixel.

**Table 2:** Posterior Summaries of Interpretable Parameters Derived from the Hierarchical Bayesian Model

Parameter	Mean	SD	2.5%	97.5%
Global means of superpixel parameters across superpixels				
Population intercept	73.05	1.91	69.31	76.77
Random intercept SD	14.84	0.81	13.39	16.56
Population slope	−0.357	0.041	−0.438	−0.277
Random slope SD	0.845	0.048	0.757	0.945
Correlation between random intercepts and slopes	−0.266	0.032	−0.328	−0.202
Mean of random residual SD	1.947	0.038	1.874	2.024
SD of random residual SD	0.741	0.031	0.682	0.805
Global SDs of superpixel parameters across superpixels				
Population intercept	13.29	1.37	10.93	16.22
Random intercept SD	5.50	0.77	4.25	7.31
Population slope	0.266	0.030	0.214	0.332
Random slope SD	0.317	0.046	0.243	0.422
Correlation between random intercepts and slopes	0.198	0.021	0.161	0.243
Mean of random residual SD	0.249	0.029	0.199	0.311
SD of random residual SD	0.171	0.029	0.122	0.235
Correlations of superpixel parameters				
Population intercept and random intercept SD	0.812	0.041	0.715	0.875
Population intercept and random slope SD	0.739	0.056	0.612	0.829
Random intercept SD and random slope SD	0.845	0.043	0.747	0.914

SD = standard deviation.

### Correlation Analyses

The correlograms for random intercepts, slopes, log residual variances, and residuals (Fig S2A–D) demonstrated that these parameters for any given superpixel tended to be most highly correlated with neighbors with decreasing correlations as a function of distance from the index superpixel. Horizontal (left–right) correlations were stronger, often far stronger at larger angular distance than vertical (up–down) correlations. Vertical correlations were strongly attenuated across the temporal raphe, but this effect was not nearly as strong along the nasal horizontal meridian. Superpixels above the horizontal median generally showed stronger correlations with other superpixels located above this median. Superpixels below the horizontal median showed lower correlations in general. The parafoveal superpixels (superpixels 4.4, 4.5, 5.4, 5.5) tended to have lower correlations with other superpixels than pairs of superpixels beyond the parafoveal area.

The strongest correlations among the 4 parameters of interest (the 3 random effects and the residuals) were seen with random intercepts (between 0.7 and 0.9 for virtually all superpixels for nearest neighbors) (Fig S2A). Correlations were high across the macula and very high between pairs of superpixels when both were located above or below the horizontal meridian.

Random slope correlations (Fig S2B) were less strong than the random intercept correlations, and the strongest correlations were only seen between neighboring superpixels and rarely at a separation of even 2 superpixels. Strongest correlation was around 0.6, and neighboring superpixels tended to have correlations ranging from about 0.3 to 0.55. Distant superpixels tended to have weaker correlations (0.1–0.2).

Correlations between the subject log residual SDs (Fig S2C) were weaker than for the random slope correlations; the strongest correlations were under 0.4, and most correlations were less than 0.3. The residual correlations (Fig S2D) were moderate between near neighbor superpixels and consistently positive though small across the entire macula. Residual correlations were smoother across the entire macula than the 3 random-effects correlations. The patterns of correlations were similar to the random slopes and log residual SDs.

### Eigenvector Analyses

**Figure 2:**
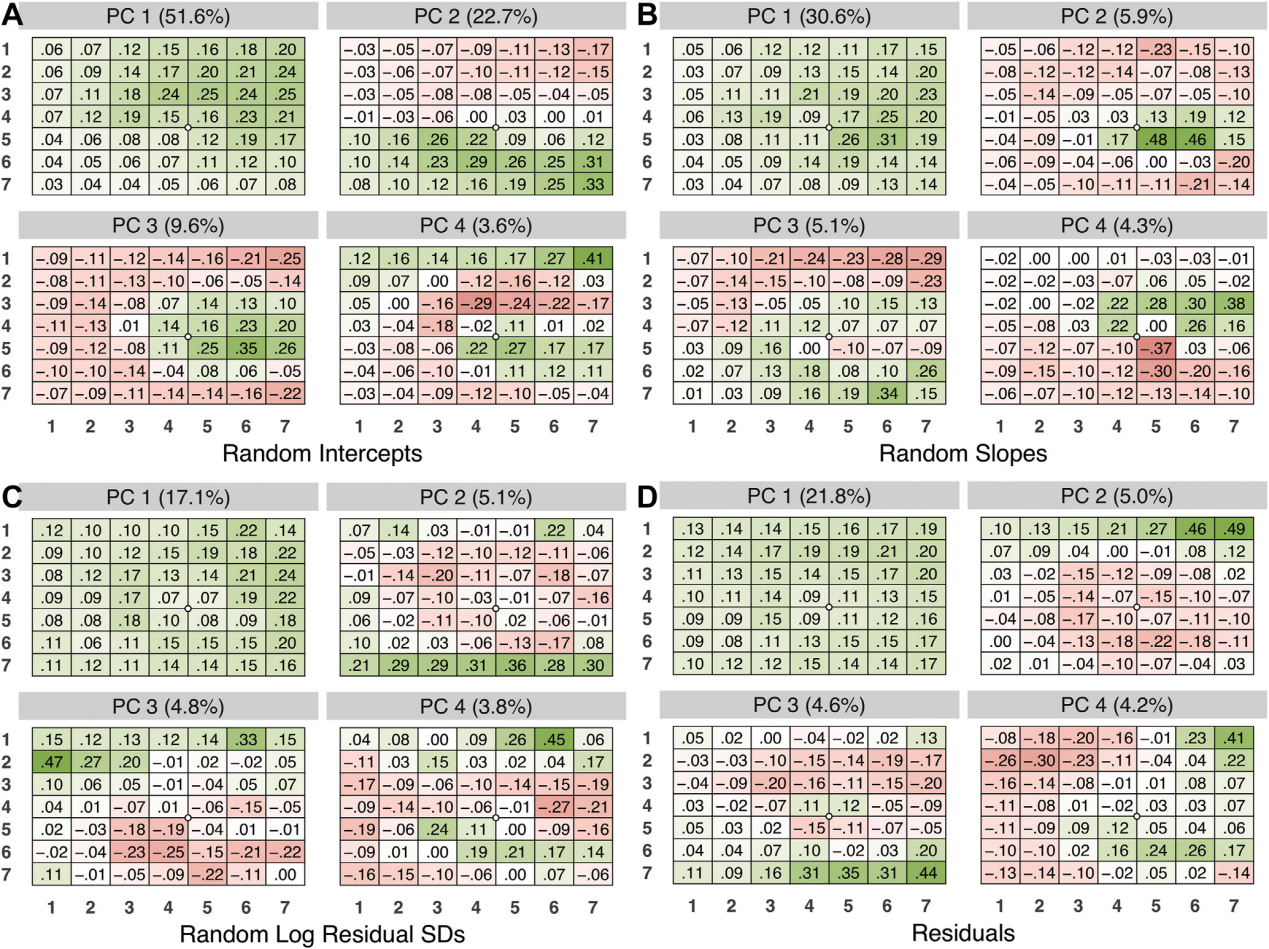
The 4 largest principal components and percent variance explained from each component of the principal component analysis of the covariances between (**A**) random intercepts, (**B**) random slopes, (**C**) random log residual SDs, and (**D**) residuals. The white circle indicates the fovea for visual orientation. SD = standard deviation.

Figure 2 plots the first 4 PCs and gives the percent of variance explained by each PC for all 4 PC decompositions. The largest PC for all 4 effects corresponds to a random global component across the macula, that is, a grand mean where within a person, the random intercepts, slopes, log residual SDs, and residuals all tend to be higher or lower together, though with somewhat different variances across the macula. The joint co-variation tended to be stronger above the horizontal meridian, weaker below, lower near the parafoveal region, and slightly lower in the periphery.

The second and third largest PCs for random intercepts and for random slopes correspond to contrasts between superior superpixels above the temporal horizontal meridian versus inferior superpixels below this meridian and a contrast between the inner/nasal macula versus the temporal and peripheral regions. For the random log residual SDs and for the residuals, the second and third eigenfunctions are contrasts between upper (lower) central superpixels and lower (upper) peripheral superpixels.

For the random intercepts, the first 3 PCs explained 51.6%, 22.7%, and 9.6% of the variation across the macula for a total of 83.9%. The first 3 eigenvalues accounted for 41.6%, 27.0%, and 31.4% of the variation in the random slopes, log residual SDs, and residuals, respectively. For the random slopes, log random residual SDs, and residuals, the first PC explained 30.6%, 17.1%, and 21.8% of the corresponding variations; the second and third components explained only about 4% to 6% of total variation each.

**Figure 3:**
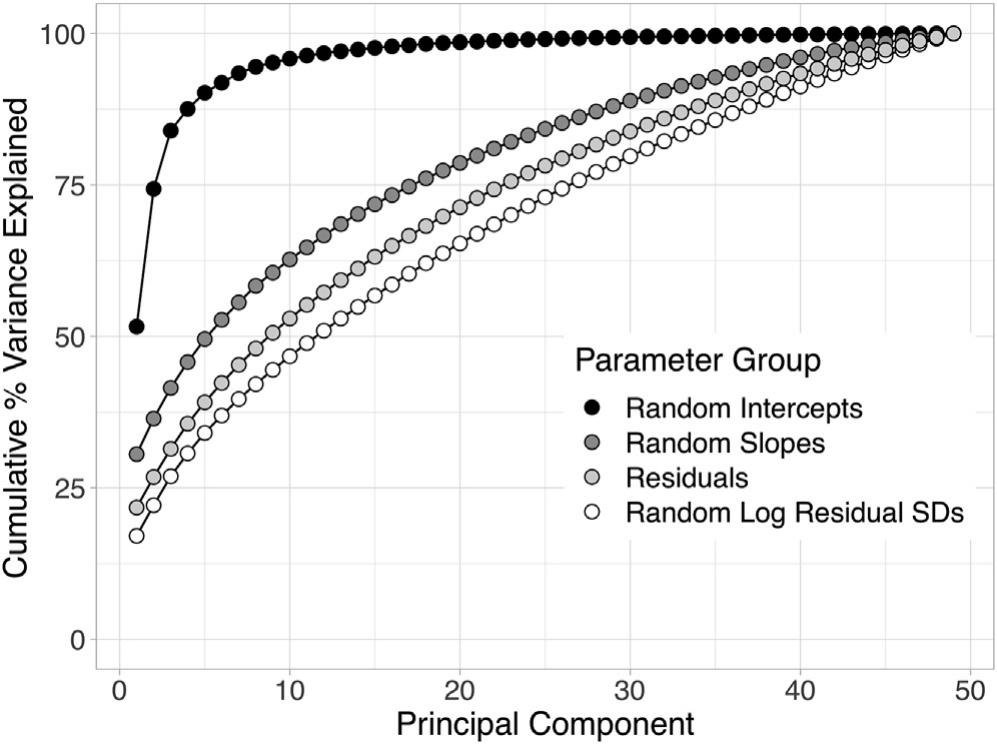
Scree plots of the cumulative percent variance explained by the principal components from the principal component analysis on the covariances.

Figure 3 provides scree plots of the cumulative percentage of variation explained by all PCs. We found similar findings using a PC analysis on the correlation (rather than covariance) matrices; the 4 largest PCs are displayed in Fig S3, and scree plots of the cumulative percentage of variation explained by PCs are shown in Fig S4.

## Discussion

We recently developed a hierarchical Bayesian random-effects model to describe longitudinal changes in thickness measurements in individual macular superpixels.^13^ Previous work in estimating structural rates of change in the macula has used simple linear regression using data from a single eye or individual sectors or superpixels to estimate an individual eye’s rate of change; such individual estimates are frequently averaged to calculate population estimates in an ad hoc fashion.^12^^,^^30^, ^31^, ^32^, ^33^, ^34^ As compared with simple linear regression, a random-effects model provides more precise estimates of individual eye rates of change using data from the entire cohort. Random-effects models incorporate population information to better estimate individual trends and model subject-specific effects more accurately in longitudinal data. Therefore, random-effects longitudinal models are preferable to simple linear regression for identifying glaucoma progression based on longitudinal macular thickness measurements.

Non-Bayesian likelihood approaches to random-effects models have difficulty with calculating standard errors for individual rates of changes and typically condition on the estimated values of uncertain hyperparameters.^35^^,^^36^ Furthermore, likelihood approaches supply standard errors based on asymptotic approximations, which may not be valid in small data sets and indeed, are not valid when random-effect variances are small. In contrast, a Bayesian approach to modeling longitudinal data provides SDs of individual and population rates of change and 95% credible intervals that fully adjust for uncertainty in all parameters and are valid when variance parameters are small.

The success of our Bayesian hierarchical random-effects models in modeling longitudinal data from a single superpixel for a cohort of glaucoma patients led us to consider whether jointly analyzing data from all the superpixels would allow for increased precision in estimating individual and population rates of change, motivating the current model. At the same time, previous research suggests that spatial similarity is not only a matter of physical adjacency but also involves the underlying biological structure of the macula.^12^^,^^22^^,^^37^ Thus, a very general spatial model such as our hierarchical random-effects model is definitely worth considering and should be in the arsenal of anyone analyzing cross-sectional or longitudinal macular thickness measures. Typical spatial models in other fields (ecology, earth science) often have only a single variable measured over space.^38^ In contrast, in our data, each eye from our cohort of 111 subjects supplies a panel of time-varying spatial data. This allows for a much more detailed and nuanced understanding of the underlying structure of the spatial components, again motivating a more general approach to spatial modeling. The random-effects spatial structure we consider here is perhaps the most general spatial structure available not incorporating any neighborhood structure, though allowing the possibility of discovering neighborhood structural patterns of correlation. This framework does not have as much power as, for example, a spatial conditional autoregressive model would have for detecting spatial structure, if that model were known to be appropriate for the data.^39^

Our current hierarchical random-effects model avoids model misspecification at the cost of statistical power potentially provided by a specific spatial model. In fact, our spatial correlational and PC analyses of the residuals and random effects show that the spatial correlations are indeed *not* well described by a neighborhood structure with a single correlation parameter as required by the popular conditional autoregressive model.

An important tool for scientific understanding is to start with simple models, identify additional modeling structure (correlations, random effects, time trends) through residual analysis, and then incorporating this structure into the next iteration of the model. One iterates again to identify further structure or confirm that the model seems satisfactory after a given step.

How to extend the original single superpixel hierarchical model to a multivariate hierarchical model is a challenging issue. There are 7 parameters for each superpixel, each of which could warrant a spatial model. At the subject level, the 3 random effects per subject (i.e., slopes, intercepts, and residual variances) can have spatial structure, and finally, residuals for each subject at each visit merit spatial modeling. To simplify this daunting task, we proposed a particular hierarchical Bayesian model for multivariate longitudinal data, in which multivariate means that we have multiple observations per subject (eye) at each visit, in particular, one observation from each of 49 superpixels. Our proposed model has 7 random effects across superpixels. This model is a major incremental step on the way to full modeling of the correlations between local macular thickness measurements at the level of superpixels. We then undertook residual spatial analysis of the random effects and residuals.

Our residual analysis extends previous Bayesian and longitudinal residual analyses.^14^, ^15^, ^16^, ^17^, ^18^, ^19^^,^^29^ We identified anatomically interpretable correlational structure in the random effects and residuals. For the random intercepts, we found extremely high correlations across the macula; thus, we expect further improvements to our model would substantially aid in estimating individual subject random intercepts. Our planned next steps in model improvement are to include the largest PC(s) identified in this paper as additional structure to this model.

The correlation analyses within our Bayesian framework (Fig S2) provided some interesting findings: The correlations decreased with increased physical distance from the superpixel of interest. However, clearly, there was a sudden drop in the correlation between adjacent superpixels at the temporal horizontal meridian. This is an expected finding given the separate course of the superior and inferior nerve fiber bundles toward the superior and inferior poles of the optic nerve head.^40^, ^41^, ^42^, ^43^ Conversely, a similar finding was not observed nasally where there is no clear-cut anatomical separation between the fiber bundles in the papillomacular region as no anatomical correlate exists for the fovea-Bruch’s membrane opening axis.^42^^,^^43^

Bayesian PC analysis across the macula provided actionable structural and modeling information. Fitting PC in a Bayesian framework allows for point estimates, SDs, and 95% credible intervals to be calculated for measures such as the percent of variation explained by a given PC. These PCs, while varying in strength from random intercepts to random slopes to random log residual SDs and residuals, showed remarkable consistency in their shape.

There is no precedent in the glaucoma OCT literature for exploration of the correlation of macular local thickness measurements. Such correlations have been investigated within the central 24° and 10° VFs.^44^ Asaoka reported that sectorization of 30-2 and 10-2 VF locations was distinct and that the identified 10-2 sectors were stable on bootstrapping.^45^ Such sectors tended to follow patterns of central RNFL bundles. This is in agreement with results reported by Nouri-Mahdavi et al^46^ based on clustering of longitudinal point-wise rates of change across 24-2 VFs.

The superpixel-specific posterior means of the 7 superpixel parameters from our multivariate model were plotted against the previously presented single superpixel modeling in Fig S1.^13^ This figure uses the same priors developed for 36 central superpixels but provides results on the 49 superpixels analyzed in this paper. The fact that the points on the 7 subplots of Fig S1 generally fall along the unity (*x* = *y*) line is mostly a testament that the prior chosen in our initial paper was adapted to mimic the priors used in this paper. The difference between the priors used in our original model and the priors here is that in the former, we identified the priors in an ad hoc way, whereas here we estimated the means and variances of the superpixel parameters in a principled fashion directly as part of the model.

In summary, we presented an innovative model for multivariate longitudinal modeling of the macular superpixels within which we estimated the global means and SDs of the superpixel-specific population parameters. PC analyses then explored underlying patterns in the random effects and residuals of the model across the macula. Introduction of subject-specific global random intercepts and slopes in addition to the superpixel-specific random effects is expected to improve estimation of both population- and subject-level intercepts and slopes. Further model enhancement may be possible by including cross-superpixel random effects and correlations to address additional spatiotemporal relationships within the macular region in the next iteration of our hierarchical framework. We plan to utilize these findings to continue to enhance the multivariate modeling of macular superpixel thickness measurements in order to provide more accurate estimates of subject and population longitudinal change over time.

## Supplementary Data

Supplemental Fig S1

Supplemental Fig S2A

Supplemental Fig S2B

Supplemental Fig S2C

Supplemental Fig S2D
