# Supplementary material for: Multivariate Longitudinal Modeling of Macular Ganglion Cell Complex: Spatiotemporal Correlations and Patterns of Longitudinal Change
Source: Ophthalmol Sci. 2022 Jun 16;2(3):100187. doi: 10.1016/j.xops.2022.100187 (PMC9559093; doi:10.1016/j.xops.2022.100187)
Supplement: Supplementary Material [file mmc1.docx]

**Supplementary Material**

**‘Multivariate Longitudinal Modeling of Macular Ganglion Cell Complex: Spatiotemporal Correlations and Patterns of Longitudinal Change’**

*Mohammadzadeh et al.*

Let $y_{\mathrm{ijk}}$ denote the GCC thickness on subject $i$ measured at the $j$th visit of subject $i$ at time $t_{ij}$ in superpixel $k$ for $k=1,\ldots, K$. Time $t_{ij}$ is measured in years, where the first visit for all subjects is at $t_{ij}=0$ years.

**Outlier Removal Algorithm for 49 Superpixels**

As described previously, we applied a semi-automated algorithm to identify and remove outliers. Prior to applying the removal algorithm, we removed all observations with GCC measurements of 0$\mu$m as zero indicates a reporting error. For each subject’s longitudinal data in each superpixel, we calculated consecutive visit absolute differences ${|y}_{ijk}-y_{i\left( j-1 \right)k}|$ and consecutive visit absolute centered slopes ${| (y}_{ijk}-y_{i\left( j-1 \right)k})/(t_{ij}-t_{i\left( j-1 \right)})+0.41|$; slopes were centered around −0.41$\mu$m/year, the mean of the pooled set of slopes from all pairs of consecutive visits across all subjects and superpixels. We flagged absolute centered slopes greater than 24 $\mu$m/year that had consecutive visit absolute differences greater than 10 $\mu$m. This value was chosen to identify approximately 0.5% of the observations as possible outliers. The flagged slope identified two consecutive points as candidates for removal. We calculated the sum of the absolute visit differences $\sum_{j=2}^{n_{ik}} |y_{ijk}- y_{i\left( j-1 \right)k}|$ for each superpixel in each subject and further considered the candidate that caused the largest decrease in the sum of the absolute visit differences. If removing the candidate resulted in a replacement absolute slope that was not one-half or less of the original flagged slope, we did not remove the observation; otherwise, we removed the candidate. If an observation was removed, we applied the same algorithm to the reduced data set to see if another observation from the same superpixel should be removed as well. For each curve, if 2 or more points were identified as outliers, we removed the entire curve. This resulted in removing 0.7% of the observations as outliers.

**Bayesian Hierarchical Longitudinal Random Intercept and Slope Model with Random Residual Variance**

We fit longitudinal data from all individuals and superpixels together in a single model. We extended our previous random intercept and slope with random residual variance model to include superpixel-specific random effects for the 7 interpretable superpixel parameters. Let $k=1, \ldots, 49$ index superpixels. The 7 interpretable parameters in superpixel $k$ are (i) population intercept $\alpha_{0k}$, (ii) standard deviation (SD) of the random intercepts $D_{00k}^{1/2}$, (iii) population slope $\alpha_{1k}$, (iv) SD of the random slopes $D_{11k}^{1/2}$, (v) correlation $\rho_{k}$ between random intercepts and slopes, (vi) mean $\sigma_{mk}$ of the random residual SDs, and (vii) SD $\sigma_{sk}$ of the random residual SDs. We use the term population here to describe parameters of the distribution of subject-specific parameters and subject data from a single superpixel. Upcoming, we use the term global for parameters of distributions that describe across-superpixel distributions of the 7 superpixel parameters.

We reparametrized this set of 7 parameters to make a multivariate normal prior more appropriate. We log transformed SD and variance parameters to reduce skewness and we transformed the (v) correlation $\rho_{k}$ and (iv) variance $D_{11k}$ of the random slopes to the regression coefficient (v)$\gamma_{k}=\rho_{k}D_{11k}^{1/2}D_{00k}^{-1/2}$ of the random slopes given the random intercept and the (iv) remaining variance $D_{11.0k}=\left( 1-\rho_{k}^{2} \right)D_{11k}$ of the random slopes (variance of the random slopes adjusted for the random intercepts). We log transformed the random intercept and slope variances (ii) $D_{00k}$, (iv) $D_{11.0k}$, and the (vi) population mean $\sigma_{mk}$ and (vii) SD $\sigma_{sk}$ of the random residual SDs.

For superpixel $k$, $\alpha_{0k}$ is the population intercept, the average intercept at time $t_{\mathrm{ij}}=0$; $\alpha_{1k}$ is the population average slope; $\beta_{0ik}$ is the $i$th subject’s random intercept: the unknown difference between subject $i$'s intercept and the population intercept $\alpha_{0k}$; $\beta_{1ik}$ is the $i$th subject’s random slope: the unknown difference between subject $i$'s slope and $\alpha_{1k}$; and the residual variance for subject $i$ is $\sigma_{ik}^{2}$. The full model is

$$y_{\mathrm{ijk}} | {\alpha_{0k}, \alpha_{1k},\beta}_{0ik},\beta_{1ik}, \sigma_{ik}^{2} \sim N({\alpha_{0k}+ \alpha_{1k}t_{\mathrm{ij}}+\beta}_{0ik}+\beta_{1ik}t_{\mathrm{ij}}, \sigma_{ik}^{2})$$

$$\beta_{0ik}|D_{00k} \sim N(0,D_{00k})$$

$$\beta_{1ik}|{\gamma_{k},\beta_{0ik},D}_{11.0k} \sim N(\gamma_{k}\beta_{0ik},D_{11.0k})$$

$$\sigma_{ik}^{-1}| \sigma_{mk},\sigma_{sk}\sim\mathrm{Gamma}(\left( \frac{\sigma_{mk}}{\sigma_{sk}} \right)^{2}+2, \frac{\sigma_{mk}^{3}}{\sigma_{sk}^{2}}+ \sigma_{mk})$$

$$\left( \alpha_{0k},\log D_{00k},\log D_{11.0k} \right)^{'} |\left( \mu_{1}, \mu_{2},\mu_{4} \right)^{'}, \Sigma\sim N(\left( \mu_{1}, \mu_{2},\mu_{4} \right)^{'}, \Sigma)$$

$$\alpha_{1k}|\mu_{3}, \sigma_{3}^{2} \sim N(\mu_{3}, \sigma_{3}^{2})$$

$$\gamma_{k}|\mu_{5}, \sigma_{5}^{2} \sim N(\mu_{5}, \sigma_{5}^{2})$$

$$\log\sigma_{mk}|\mu_{6}, \sigma_{6}^{2} \sim N(\mu_{6}, \sigma_{6}^{2})$$

$$\log\sigma_{sk}|\mu_{7}, \sigma_{7}^{2} \sim N(\mu_{7}, \sigma_{7}^{2})$$

Matrix $D_{k}$ is a 2x2 variance-covariance matrix of the random intercepts and slopes with elements

$$D_{k}=\left( \begin{aligned} D_{00k} D_{01k} \\ D_{10k}D_{11k} \end{aligned} \right)$$

and $D_{11.0k}=D_{11k}-D_{10k}D_{00k}^{-1}D_{01k}$ is the variance of the conditional distribution of $\beta_{1ik}|\beta_{0ik}$. We are particularly interested in the correlation

$$\rho_{k}= \frac{D_{01k}}{\left( D_{00k} D_{11k} \right)^{1/2}}=\gamma_{k}\sqrt{\frac{D_{00k}}{D_{11k}}}$$

between the random intercepts and slopes. The priors used are

$$\mu_{1} \sim N(90, 400)$$

$$\mu_{2} \sim N(5.4161, 0.804719)$$

$$\mu_{3} \sim N(-0.8, 0.36)$$

$$\mu_{4} \sim N(-0.4462871, 0.804719)$$

$$\mu_{5} \sim N(0, 9e-04)$$

$$\mu_{6} \sim N(0.7, 0.09)$$

$$\mu_{7} \sim N(-0.25, 0.09)$$

$$\Sigma^{-1} \sim Wish(5V, 5)$$

$$V=\left( \begin{matrix} 135 & 0 & 0 \\ 0 & 0.15 & 0 \\ 0 & 0 & 0.384 \end{matrix} \right)$$

$$\sigma_{3}^{2} \sim\mathrm{IG}(2.5, 0.1666667)$$

$$\sigma_{5}^{2} \sim\mathrm{IG}(2.5, 0.00135)$$

$$\sigma_{6}^{2} \sim\mathrm{IG}(2.5, 0.06)$$

$\sigma_{7}^{2} \sim\mathrm{IG}(2.5, 0.135)$.

**Transformation of Parameters**

We back-transformed the 7 transformed model parameters back into the 7 interpretable parameters. We did not need to back-transform the (i) population intercept $\alpha_{0k}$ or (iii) population slope $\alpha_{1k}$ since they were not transformed for modeling. For the log transformed (vi) mean $\sigma_{mk}$ and (vii) SD $\sigma_{sk}$ of the random residual SDs, we back-transformed the global mean and SD parameters $\mu_{\mathcal{l}}$ and $\sigma_{\mathcal{l}}$ using the relationships between means and variances of normal and lognormal distributions. If $\log\left( x \right)\sim N(\mu_{\mathcal{l}},\sigma_{\mathcal{l}}^{2})$, then $x \sim\mathrm{Lognormal}(\mu_{x},\sigma_{x}^{2})$, where

$\mu_{x}=exp(\mu_{\mathcal{l}}+\frac{1}{2}\sigma_{\mathcal{l}}^{2})$ (A.1)

$\sigma_{x}^{2}=(\exp\left( \sigma_{\mathcal{l}}^{2})-1 \right)exp(2\mu_{\mathcal{l}}+\sigma_{\mathcal{l}}^{2})$, (A.2)

where we parameterize the density of x by its mean $\mu_{x}$ and variance $\sigma_{x}^{2}$ rather than the more traditional mean $\mu_{\mathcal{l}}$ and variance $\sigma_{\mathcal{l}}^{2}$ of $\log(x)$. Using (A.1) and (A.2), we transform posterior samples of $\mu_{\mathcal{l}}$ and $\sigma_{\mathcal{l}}$ back to the interpretable scale. To back-transform the remaining 3 parameters (ii) variance of the random intercepts $D_{00k}$, (iv) variance of the random slopes $D_{11k}$, and (v) correlation $\rho_{k}$ between random intercepts and slopes, and to calculate correlations between parameters, we used each posterior sample of the $\mu_{\mathcal{l}}$ and $\sigma_{\mathcal{l}}$ or covariance matrix $\Sigma_{\mathcal{l}}$ to sample 16,500 superpixel random effects in addition to the original 49. Let $\phi_{\mathcal{l,}k}$ be the sample of $k$ = 1, …, 16,549 superpixel random effects for $\mathcal{l}$ = 1, …, 7 parameters. We back-transform each $\phi_{\mathcal{l,}k}$ using the following equations

$D_{00k}^{*}=exp(\phi_{2,k})$ (A.4)

$D_{11k}^{*}=\exp\left( \phi_{4,k} \right)+\phi_{5,k}D_{00k}^{*}$ (A.4)

$\rho_{k}^{*}=\phi_{5,k}\sqrt{\frac{D_{00k}^{*}}{D_{11k}^{*}}}$ (A.5)

and then estimate the global mean and SD of the back-transformed samples of $D_{00k}^{*}$, $D_{11k}^{*}$, and $\rho_{k}^{*}$. We calculate correlations between parameters using the back-transformed samples of $D_{00k}^{*}$, $D_{11k}^{*}$, and $\alpha_{0k}^{*}=\phi_{1,k}$. We chose to sample an additional 16,500 superpixel random effects so that the within sample variance (variation due to differences within individual samples) was 1% of the between sample variance (variation due to differences between sample means).

**JAGS Code**

The following JAGS code encodes our analysis model.

# JAGS model

model <- function() {

# Specify likelihood

for(i in 1:n){

y[i] ~ dnorm(theta[i], tau.e[id[i], sp[i]]^2)

theta[i] = b0[id[i], sp[i]] + (b1[id[i], sp[i]] * fuyrs[i])

}

# Specify priors

for (k in 1:n_sp) { # k loops over 49 superpixels

# mu_matrix: rows are random intercept, log D00, and log D11.0

# and columns are superpixels.

mu_matrix[1:3, k] ~ dmnorm(mu[1:3], invSigma)

# a1: random slope

a1[k] ~ dnorm(a1_mu, a1_tau)

# log_mean: mean of the random residual SDs

log_mean[k] ~ dnorm(log_mean_mu, log_mean_tau)

# log_sd: SD of the random residual SDs

log_sd[k] ~ dnorm(log_sd_mu, log_sd_tau)

# tau.a: shape parameter for IG prior on random residual SD

tau.a[k] = (exp(log_mean[k]) / exp(log_sd[k]))^2 + 2

# tau.b: scale parameter for IG prior on random residual SD

tau.b[k] = (exp(log_mean[k])^3 / exp(log_sd[k])^2) +

exp(log_mean[k])

# gamma: regression coefficient

gamma[k] ~ dnorm(gamma_mu, gamma_tau)

for (j in 1:n_id) { # j loops over 111 subjects

# b0: subject specific intercept

b0[j, k] ~ dnorm(mu_matrix[1, k], 1 / exp(mu_matrix[2, k]))

# b1: subject specific slope

b1[j, k] ~ dnorm(a1[k] + gamma[k] *

(b0[j, k] - mu_matrix[1, k]),

1 / exp(mu_matrix[3, k]))

# tau.e: 1 / subject specific residual SD

tau.e[j, k] ~ dgamma(tau.a[k], tau.b[k])

}

}

# Priors for parameters

invSigma ~ dwish(V, 5) # V is passed into JAGS model

mu[1] ~ dnorm(90, 1 / 400)

mu[2] ~ dnorm(5.4161, 1 / 0.804719)

mu[3] ~ dnorm(-0.4462871, 1 / 0.804719)

a1_mu ~ dnorm(-0.8, 1 / 0.36)

a1_tau ~ dgamma(2.5, 0.1666667)

gamma_mu ~ dnorm(0, 1 / 9e-04)

gamma_tau ~ dgamma(2.5, 0.00135)

log_mean_mu ~ dnorm(0.7, 1 / 0.09)

log_mean_tau ~ dgamma(2.5, 0.06)

log_sd_mu ~ dnorm(-0.25, 1 / 0.09)

log_sd_tau ~ dgamma(2.5, 0.135)

}

V = 5 * diag(c(135, 0.150, 0.384)) # to be passed into JAGS model

**Time Series Plots**

eFigure 5 presents time series plots of the Markov chain Monte Carlo samples for a selection of parameters. These plots and those for other (not shown) parameters, illustrated satisfactory convergence.

**Correlations and Principal Components Analysis**

Using our Bayesian model, we are able to estimate correlations between pairs of superpixels of subject-specific random intercepts $\beta_{0ik}$, random slopes $\beta_{1ik}$, random log residual SDs $\log\sigma_{ik}$, as well as observation-level residuals ($y_{ijk}-({\alpha_{0k}+ \alpha_{1k}t_{\mathrm{ij}}+\beta}_{0ik}+\beta_{1ik}t_{\mathrm{ij}})$). We calculated correlations using all complete pairs of parameter estimates between pairs of superpixels, since we removed outlying points and curves from some superpixels during the outlier removal process. For each Markov Chain Monte Carlo (MCMC) sample from the posterior, we calculated a 49 × 49 correlation matrix and then averaged each matrix element over the 9,000 iterations. We refer to the estimated correlation matrix as the averaged correlation matrix. We then plotted these averaged 49 × 49 correlation matrices to visualize the correlations (see eFigure 2a-d). Similarly, at each MCMC iteration, we also calculated a 49 × 49 covariance matrix and then averaged each matrix element over the 9,000 iterations.

We applied principal components analysis (PCA) to the averaged covariance and correlation matrices across superpixels of the (i) subject-specific random intercepts, (ii) random intercepts, (iii) random log residual SDs, and (iv) observation-level residuals. When PCA is applied to the correlation matrix, the 49 variables are standardized, and the total variance will be equal to the number of variables (superpixels). When PCA is applied to the covariance matrix, the variables are not standardized. The principal components decompositions factorize the correlation or covariance matrix $A=V\Lambda V^{'}$ where $V$ is an orthogonal matrix with $VV’ = V’V = I$, and I is the identity matrix. Then $V$ is a square matrix with 49 linearly independent eigenvectors whose $k$^th^ column is the *k*^th^ eigenvector and $\Lambda$ is a diagonal matrix whose $k$^th^ diagonal element is the eigenvalue corresponding to the *k*^th^ eigenvector. The eigenvalues are the variance explained by the corresponding eigenvectors (principal components). We plotted the top 4 principal components from the principal components analysis of the covariances in Figure 2 and of the correlations in eFigure 3.

When the multivariate measures are repeated measures then principal components on the covariance matrix is preferred. Our multivariate data is GCC thickness in microns, a repeated measure over space that is comparable from one superpixel to the next. In contrast, when the multivariate measures are non-commensurate, such as if the multivariate measures were heart rate, systolic blood pressure and a cholesterol reading, then only the correlation matrix version of principal components typically makes sense. We illustrate both in this paper, results are generally similar, though not identical.

**eFigure Legends**

**eFigure 1**. Scatterplots comparing the estimates of the 7 superpixel parameters from fitting each superpixel separately (Separate Models) and all superpixels simultaneously in one model (One Model). The 7 interpretable parameters are a) population intercept, b) SD of random intercepts, c) population slope, d) SD of random slopes, e) correlation between random intercepts and slopes, f) mean of the random residual SD, and g) SD of the random residual SD. The loess curve and x=y identity line is shown in blue and red, respectively.

**eFigure 2a**. Estimated correlations between the subject-specific random intercept in each superpixel and that in the other superpixels across the macular region. Each superpixel map shows the pairwise correlations between the labeled superpixel and the other 48 superpixels. The black box indicates the location of the labeled superpixel. The white circle indicates the fovea for visual orientation.

**eFigure 2b**. Estimated correlations between the subject-specific random slope in each superpixel and that in the other superpixels across the macular region. Each superpixel map shows the pairwise correlations between the labeled superpixel and the other 48 superpixels. The black box indicates the location of the labeled superpixel. The white circle indicates the fovea for visual orientation.

**eFigure 2c**. Estimated correlations between the subject-specific random log residual SD at each superpixel and that in all the other superpixels across the macular region. Each superpixel map shows the pairwise correlations between the labeled superpixel and all the other 48 superpixels. The black box indicates the location of the labeled superpixel. The white circle indicates the fovea for visual orientation.

**eFigure 2d**. Estimated correlations between subject-observation residual in each superpixel and that in all other superpixels. Each superpixel map shows the pairwise correlations between the labeled superpixel and the other 48 superpixels. The black box indicates the location of the labeled superpixel. The white circle indicates the fovea for visual orientation.

**eFigure 3**. The 4 largest principal components and percent variance explained from each component from the principal components analysis of the correlations between a) random intercepts, b) random slopes, c) random log residual SDs, and d) residuals. The white circle indicates the fovea for visual orientation.

**eFigure 4**. Scree plot of the cumulative percent of variance explained by the principal components from the principal components analysis on the correlation matrices.

**eFigure 5.** Time series plots of the Markov chain Monte Carlo samples for a selection of global, superpixel, and subject-level parameters.
