# Supplementary figures and images for: Multivariate Longitudinal Modeling of Macular Ganglion Cell Complex: Spatiotemporal Correlations and Patterns of Longitudinal Change
Source: Ophthalmol Sci. 2022 Jun 16;2(3):100187. doi: 10.1016/j.xops.2022.100187 (PMC9559093; doi:10.1016/j.xops.2022.100187)

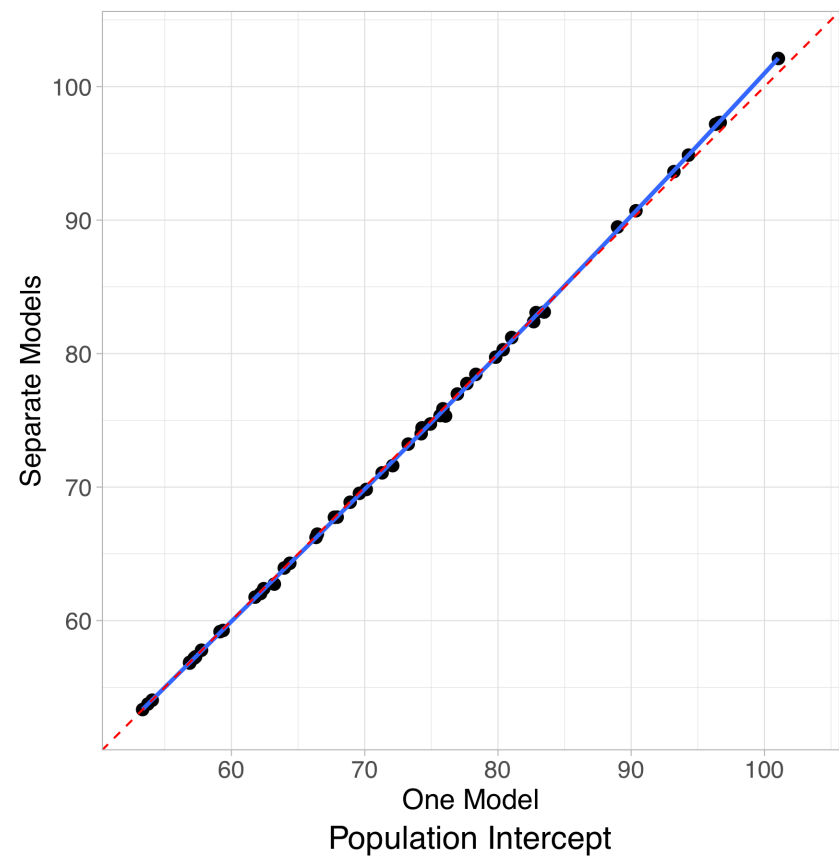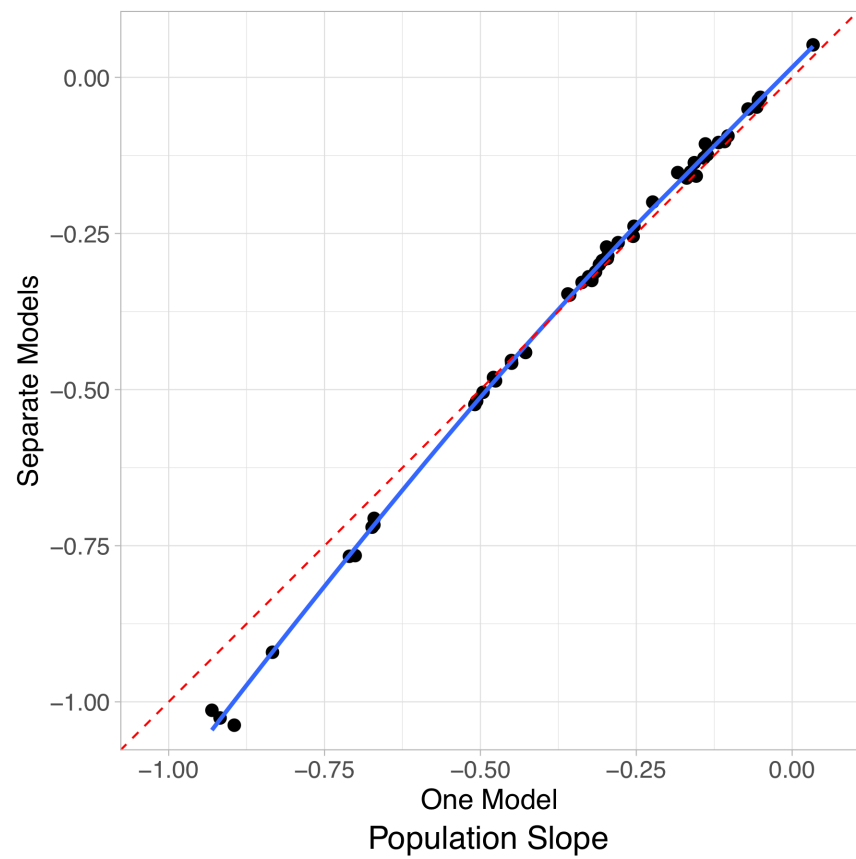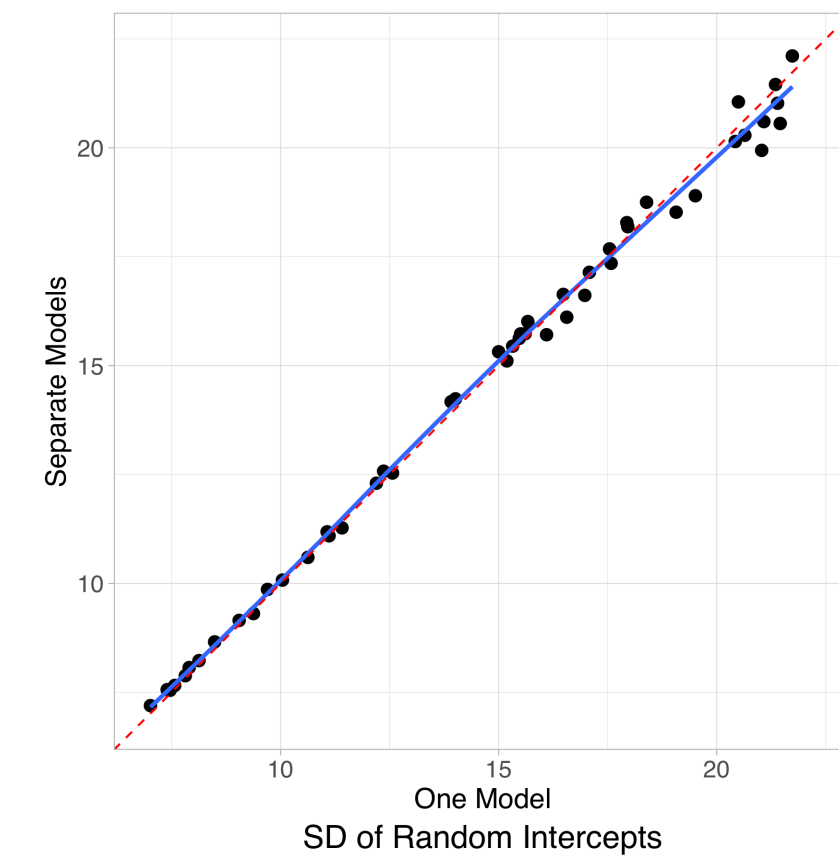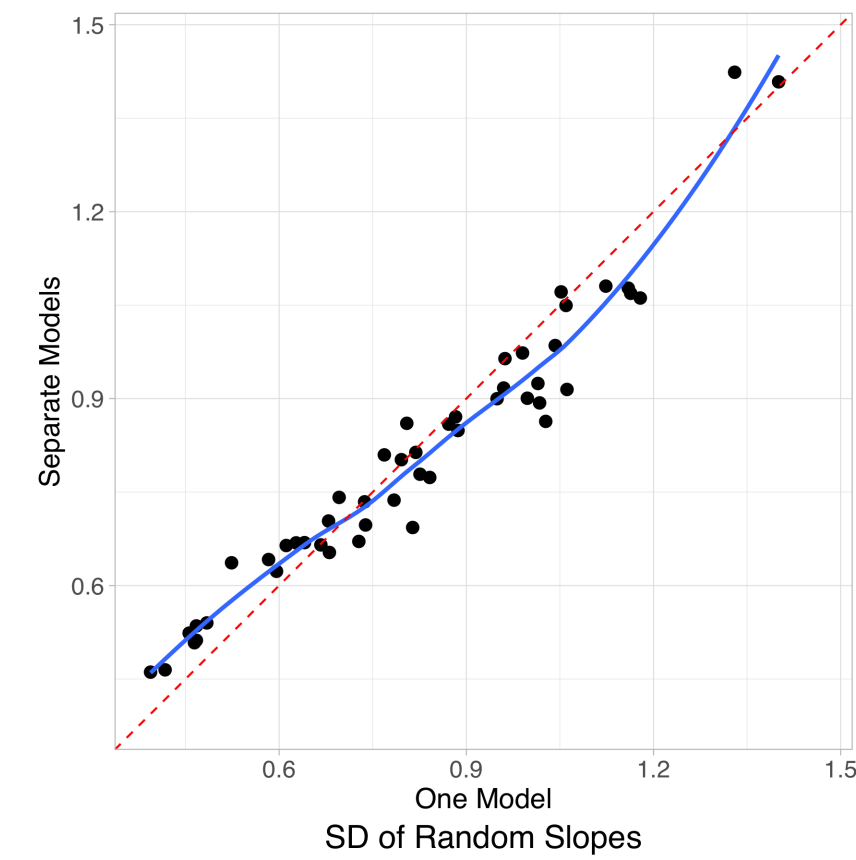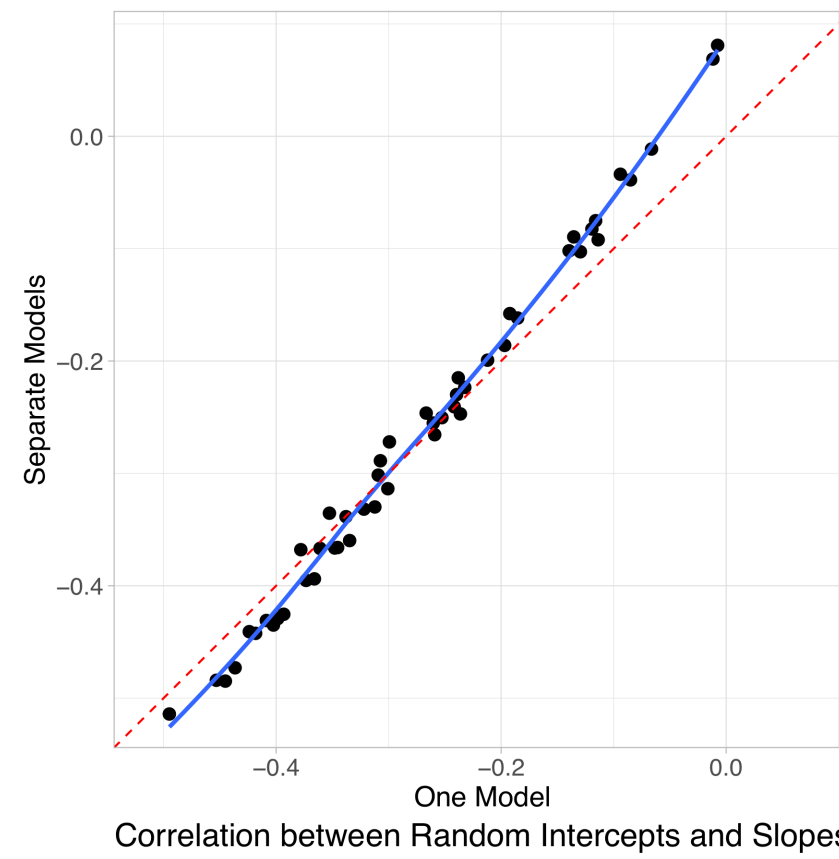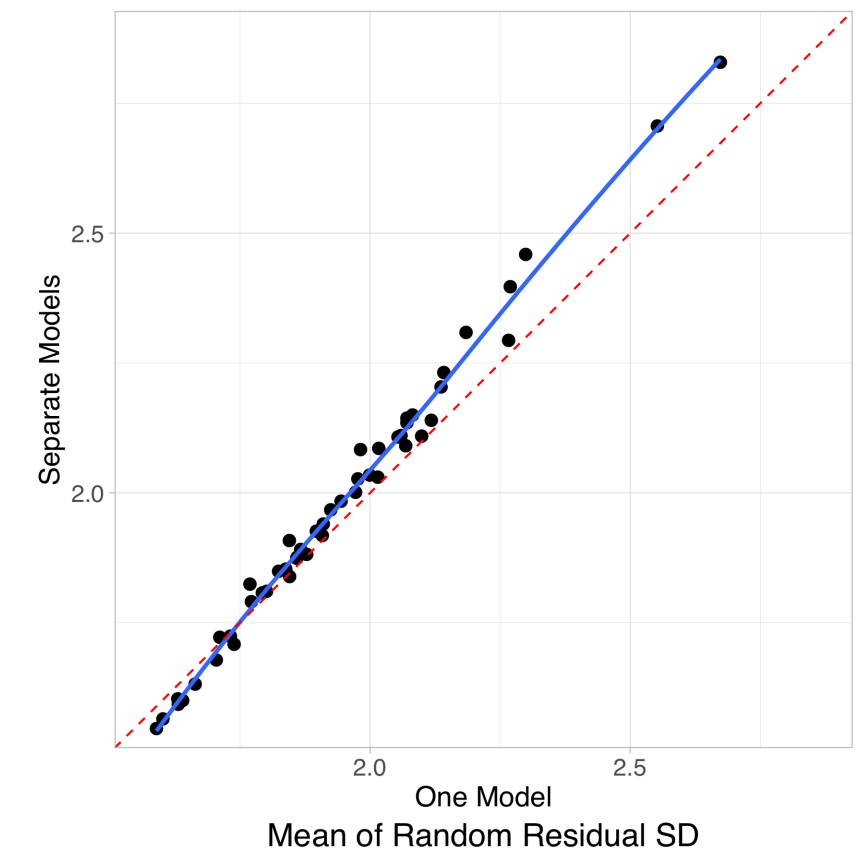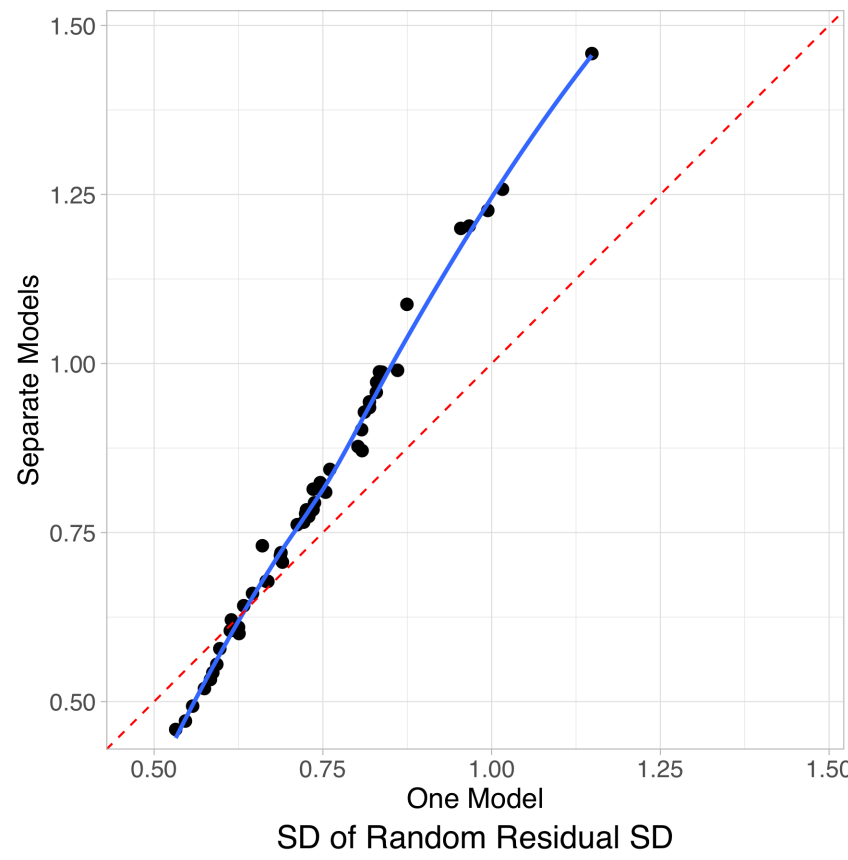

Supplement: Supplemental Fig S1 [file mmc2.pdf]

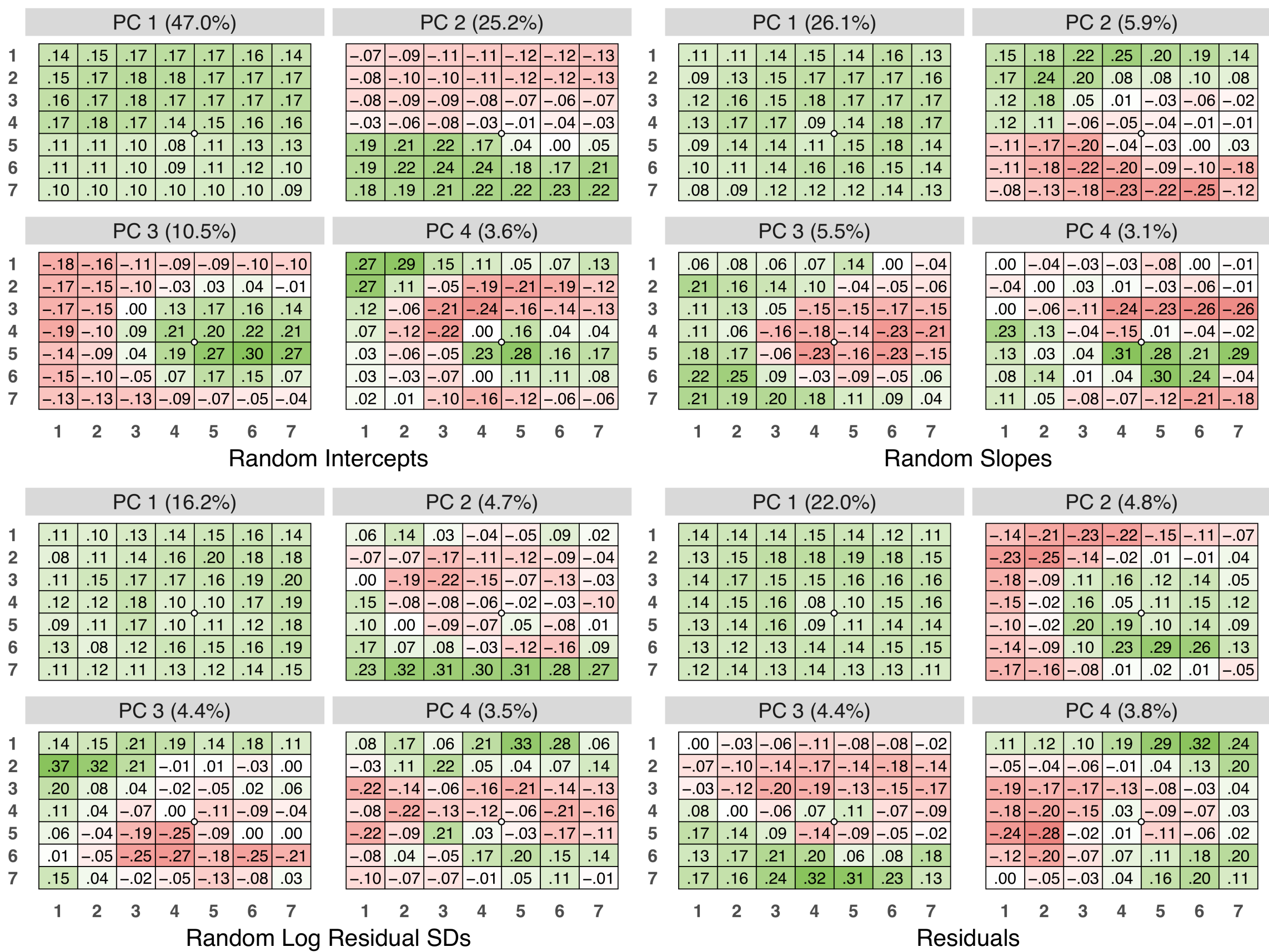

Supplement: Supplemental Fig S3 [file mmc7.pdf]

Cumulative % Variance Explained

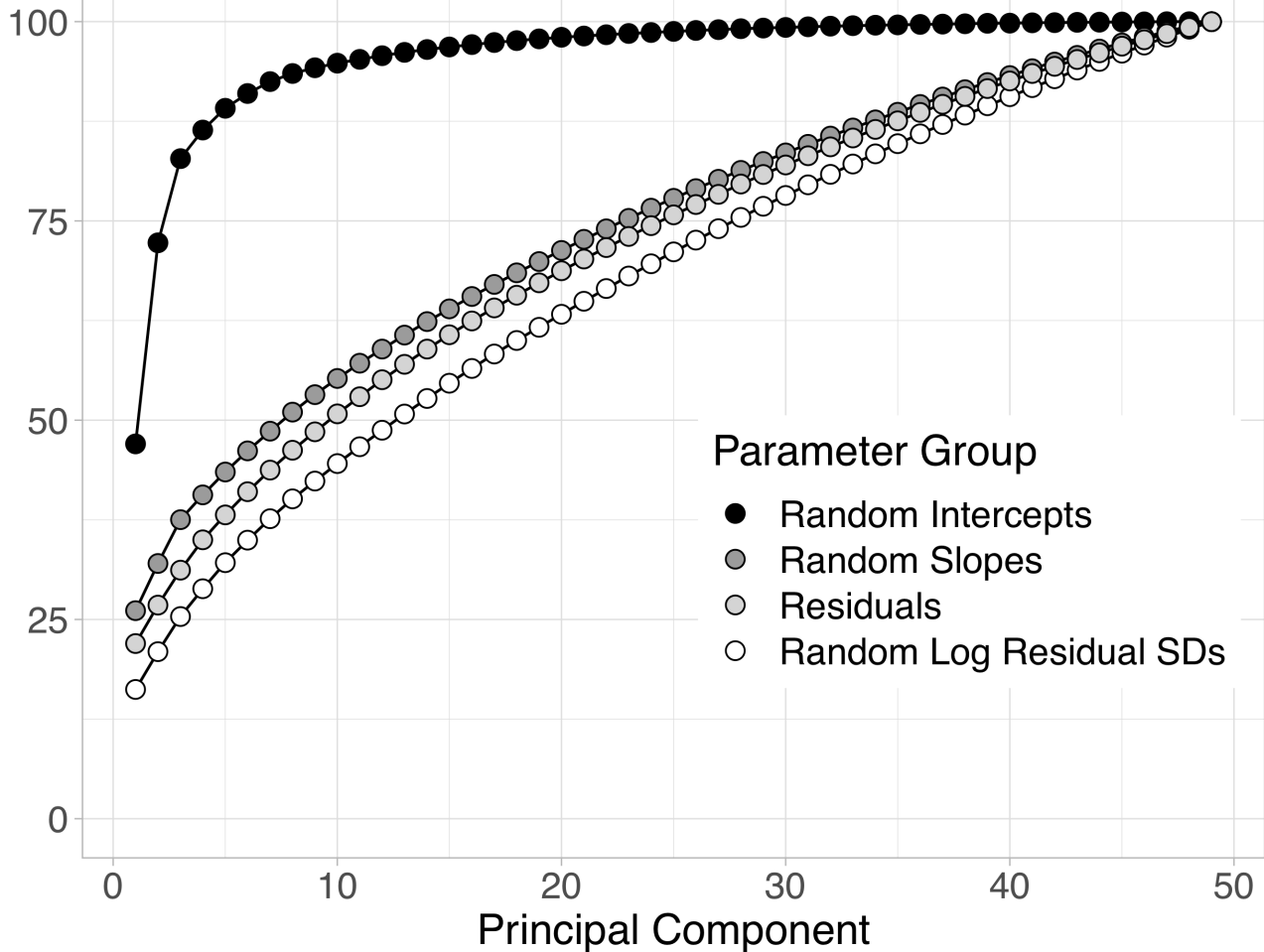

Supplement: Supplemental Fig S4 [file mmc8.pdf]

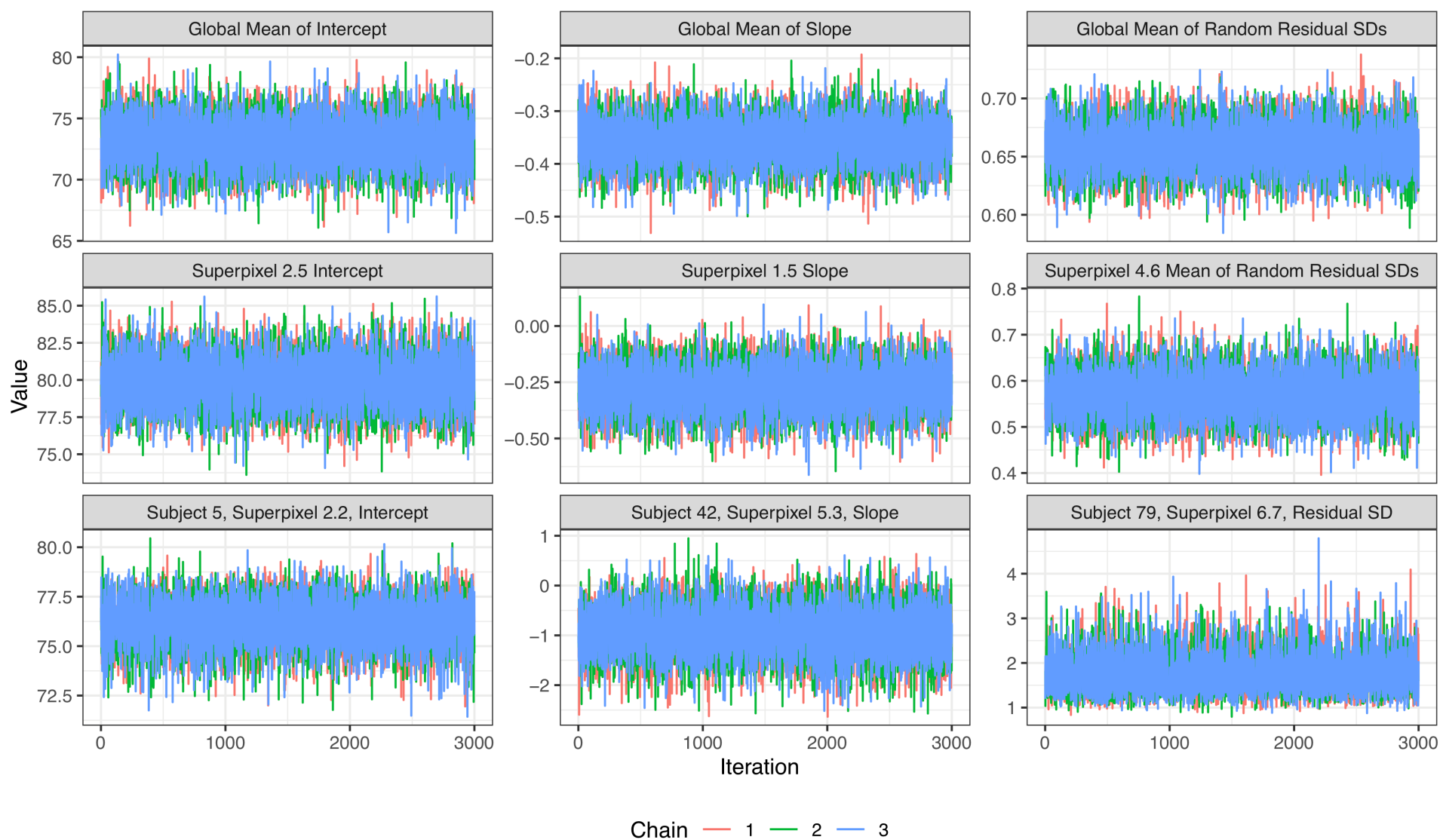

Supplement: Supplemental Fig S5 [file mmc9.pdf]
