# Supplementary material for: Multivariate Longitudinal Modeling of Macular Ganglion Cell Complex: Spatiotemporal Correlations and Patterns of Longitudinal Change
Source: Ophthalmol Sci. 2022 Jun 16;2(3):100187. doi: 10.1016/j.xops.2022.100187 (PMC9559093; doi:10.1016/j.xops.2022.100187)
Supplement: Supplemental Fig S2A [file mmc3.pdf]

1.1

	.88	.81	.79	.76	.76	.75
.83	.81	.73	.62	.56	.57	.64
.80	.75	.57	.42	.41	.43	.47
.75	.67	.45	.34	.39	.39	.39
.31	.23	.05	.03	.19	.22	.22
.36	.25	.12	.00	.07	.12	.10
.31	.32	.20	.14	.15	.14	.10

1.2

.88		.89	.86	.80	.79	.77
.86	.88	.77	.65	.60	.61	.67
.83	.79	.61	.47	.45	.47	.51
.77	.70	.49	.35	.40	.41	.42
.27	.18	.02	.03	.22	.25	.26
.30	.20	.09	−.01	.10	.16	.11
.24	.23	.14	.10	.11	.10	.05

1.3

.81	.89		.94	.89	.85	.79
.81	.90	.86	.77	.74	.74	.78
.82	.84	.72	.64	.63	.64	.67
.76	.76	.63	.48	.48	.55	.55
.24	.18	.03	.04	.26	.34	.32
.27	.17	.08	−.01	.12	.20	.13
.23	.20	.12	.12	.12	.11	.07

1.4

.79	.86	.94		.96	.91	.86
.80	.91	.91	.84	.80	.81	.85
.83	.88	.79	.69	.67	.67	.70
.77	.82	.69	.50	.50	.58	.59
.22	.18	.04	.05	.29	.39	.36
.24	.16	.08	−.01	.13	.20	.13
.20	.18	.12	.12	.12	.11	.08

1.5

.76	.80	.89	.96		.95	.87
.75	.87	.89	.85	.82	.83	.89
.79	.86	.79	.69	.66	.65	.70
.74	.82	.71	.50	.47	.56	.57
.17	.15	.05	.03	.25	.37	.34
.19	.13	.07	−.01	.11	.17	.11
.17	.14	.11	.11	.11	.10	.08

1.6

.76	.79	.85	.91	.95		.92
.72	.81	.83	.78	.75	.78	.87
.73	.82	.72	.62	.59	.59	.64
.69	.77	.65	.49	.44	.51	.52
.11	.11	.03	−.01	.21	.32	.29
.12	.07	.04	−.03	.07	.13	.08
.12	.09	.08	.09	.10	.07	.08

1.7

.75	.77	.79	.86	.87	.92	
.70	.77	.75	.69	.67	.70	.80
.71	.78	.65	.55	.53	.52	.58
.68	.72	.57	.42	.43	.45	.46
.08	.06	−.03	−.03	.21	.27	.22
.08	.03	−.03	−.08	.02	.07	.02
.04	.02	.01	.02	.04	.01	.05

2.1

.83	.86	.81	.80	.75	.72	.70
	.86	.76	.64	.59	.59	.60
.87	.76	.61	.48	.47	.48	.50
.82	.69	.49	.36	.41	.42	.44
.37	.25	.08	.04	.20	.28	.28
.38	.26	.14	.03	.10	.16	.09
.32	.30	.19	.12	.13	.09	.04

2.2

.81	.88	.90	.91	.87	.81	.77
.86		.90	.82	.77	.76	.77
.90	.91	.77	.64	.60	.61	.63
.84	.82	.64	.40	.44	.50	.52
.32	.24	.07	.02	.21	.31	.30
.33	.23	.11	.01	.12	.19	.11
.26	.24	.17	.15	.15	.10	.06

2.3

.73	.77	.86	.91	.89	.83	.75
.76	.90		.94	.89	.87	.85
.85	.93	.89	.76	.72	.70	.71
.79	.89	.77	.50	.48	.58	.58
.29	.26	.12	.06	.26	.38	.35
.32	.24	.15	.05	.16	.22	.14
.29	.25	.20	.20	.18	.15	.10

2.4

.62	.65	.77	.84	.85	.78	.69
.64	.82	.94		.97	.94	.90
.77	.88	.96	.86	.79	.77	.78
.71	.88	.87	.53	.49	.63	.63
.21	.21	.09	.04	.29	.45	.41
.23	.17	.10	.03	.16	.21	.10
.20	.18	.15	.16	.15	.10	.05

2.5

.56	.60	.74	.80	.82	.75	.67
.59	.77	.89	.97		.98	.92
.70	.83	.95	.91	.85	.82	.84
.65	.84	.90	.59	.52	.67	.68
.14	.15	.08	.06	.33	.52	.48
.16	.12	.06	.04	.19	.25	.11
.12	.12	.10	.13	.13	.07	.03

2.6

.57	.61	.74	.81	.83	.78	.70
.59	.76	.87	.94	.98		.95
.68	.81	.92	.89	.84	.82	.85
.62	.82	.88	.60	.52	.68	.70
.12	.13	.08	.07	.33	.53	.50
.12	.10	.05	.04	.20	.27	.13
.11	.10	.08	.11	.12	.07	.03

2.7

.64	.67	.78	.85	.89	.87	.80
.60	.77	.85	.90	.92	.95	
.68	.82	.86	.80	.75	.74	.79
.63	.80	.81	.54	.48	.62	.65
.07	.09	.04	.03	.28	.45	.42
.09	.06	.02	.00	.15	.21	.10
.07	.07	.06	.10	.11	.06	.06

3.1

.80	.83	.82	.83	.79	.73	.71
.87	.90	.85	.77	.70	.68	.68
	.89	.74	.56	.53	.54	.57
.88	.80	.59	.34	.42	.46	.47
.38	.28	.09	.03	.18	.26	.27
.39	.27	.15	.04	.12	.18	.12
.32	.30	.24	.20	.19	.13	.07

3.2

.75	.79	.84	.88	.86	.82	.78
.76	.91	.93	.88	.83	.81	.82
.89		.87	.70	.64	.62	.65
.85	.93	.73	.42	.41	.49	.50
.34	.31	.14	.03	.17	.27	.27
.33	.27	.18	.06	.14	.20	.13
.26	.25	.24	.25	.23	.16	.14

3.3

.57	.61	.72	.79	.79	.72	.65
.61	.77	.89	.96	.95	.92	.86
.74	.87		.90	.82	.79	.79
.71	.89	.92	.58	.52	.64	.63
.25	.25	.17	.11	.33	.48	.45
.23	.20	.15	.10	.24	.28	.15
.21	.18	.17	.21	.19	.13	.07

3.4

.42	.47	.64	.69	.69	.62	.55
.48	.64	.76	.86	.91	.89	.80
.56	.70	.90		.95	.91	.90
.54	.75	.93	.75	.63	.76	.75
.17	.21	.20	.18	.45	.64	.58
.16	.17	.14	.15	.31	.35	.19
.13	.11	.10	.16	.16	.12	.08

3.5

.41	.45	.63	.67	.66	.59	.53
.47	.60	.72	.79	.85	.84	.75
.53	.64	.82	.95		.98	.95
.50	.68	.84	.79	.74	.87	.86
.17	.22	.19	.22	.53	.73	.66
.18	.21	.16	.18	.35	.38	.22
.16	.16	.14	.17	.18	.13	.12

3.6

.43	.47	.64	.67	.65	.59	.52
.48	.61	.70	.77	.82	.82	.74
.54	.62	.79	.91	.98		.97
.50	.65	.79	.75	.74	.88	.88
.19	.23	.20	.22	.51	.72	.68
.22	.24	.19	.20	.36	.41	.26
.19	.20	.19	.20	.22	.17	.15

3.7

.47	.51	.67	.70	.70	.64	.58
.50	.63	.71	.78	.84	.85	.79
.57	.65	.79	.90	.95	.97	
.52	.67	.80	.75	.73	.85	.88
.18	.21	.19	.20	.49	.69	.66
.20	.22	.17	.18	.34	.39	.26
.17	.19	.18	.21	.24	.17	.15

4.1

.75	.77	.76	.77	.74	.69	.68
.82	.84	.79	.71	.65	.62	.63
.88	.85	.71	.54	.50	.50	.52
	.86	.60	.36	.42	.41	.40
.56	.48	.26	.12	.18	.21	.24
.51	.44	.32	.18	.19	.25	.22
.41	.42	.38	.36	.35	.27	.22

4.2

.67	.70	.76	.82	.82	.77	.72
.69	.82	.89	.88	.84	.82	.80
.80	.93	.89	.75	.68	.65	.67
.86		.82	.51	.49	.54	.52
.42	.43	.27	.13	.25	.34	.34
.36	.35	.28	.17	.23	.27	.20
.31	.30	.30	.32	.31	.24	.21

4.3

.45	.49	.63	.69	.71	.65	.57
.49	.64	.77	.87	.90	.88	.81
.59	.73	.92	.93	.84	.79	.80
.60	.82		.73	.60	.69	.66
.21	.26	.26	.23	.44	.59	.53
.14	.17	.16	.17	.32	.34	.19
.14	.11	.12	.19	.18	.12	.07

4.4

.34	.35	.48	.50	.50	.49	.42
.36	.40	.50	.53	.59	.60	.54
.34	.42	.58	.75	.79	.75	.75
.36	.51	.73		.82	.76	.71
.15	.24	.31	.41	.63	.70	.59
.12	.18	.19	.24	.36	.37	.26
.15	.12	.09	.14	.16	.18	.19

4.5

.39	.40	.48	.50	.47	.44	.43
.41	.44	.48	.49	.52	.52	.48
.42	.41	.52	.63	.74	.74	.73
.42	.49	.60	.82		.88	.82
.26	.30	.30	.46	.76	.75	.65
.24	.29	.24	.28	.42	.43	.32
.23	.26	.18	.19	.22	.25	.26

4.6

.39	.41	.55	.58	.56	.51	.45
.42	.50	.58	.63	.67	.68	.62
.46	.49	.64	.76	.87	.88	.85
.41	.54	.69	.76	.88		.96
.19	.24	.26	.36	.67	.83	.75
.21	.25	.21	.26	.43	.46	.30
.20	.22	.16	.17	.20	.19	.17

4.7

.39	.42	.55	.59	.57	.52	.46
.44	.52	.58	.63	.68	.70	.65
.47	.50	.63	.75	.86	.88	.88
.40	.52	.66	.71	.82	.96	
.20	.23	.25	.35	.64	.84	.79
.23	.25	.22	.27	.45	.50	.34
.21	.24	.18	.19	.23	.20	.19

5.1

.31	.27	.24	.22	.17	.11	.08
.37	.32	.29	.21	.14	.12	.07
.38	.34	.25	.17	.17	.19	.18
.56	.42	.21	.15	.26	.19	.20
	.91	.75	.47	.22	.15	.30
.90	.91	.84	.70	.58	.59	.67
.80	.83	.84	.82	.79	.76	.70

5.2

.23	.18	.18	.18	.15	.11	.06
.25	.24	.26	.21	.15	.13	.09
.28	.31	.25	.21	.22	.23	.21
.48	.43	.26	.24	.30	.24	.23
.91		.89	.56	.24	.18	.32
.85	.93	.94	.81	.65	.64	.74
.75	.80	.84	.88	.86	.84	.81

5.3

.05	.02	.03	.04	.05	.03	−.03
.08	.07	.12	.09	.08	.08	.04
.09	.14	.17	.20	.19	.20	.19
.26	.27	.26	.31	.30	.26	.25
.75	.89		.74	.34	.29	.43
.67	.79	.92	.94	.80	.77	.84
.61	.63	.71	.82	.84	.83	.78

5.4

.03	.03	.04	.05	.03	−.01	−.03
.04	.02	.06	.04	.06	.07	.03
.03	.03	.11	.18	.22	.22	.20
.12	.13	.23	.41	.46	.36	.35
.47	.56	.74		.68	.55	.66
.41	.51	.58	.79	.87	.83	.79
.39	.40	.41	.49	.54	.57	.55

5.5

.19	.22	.26	.29	.25	.21	.21
.20	.21	.26	.29	.33	.33	.28
.18	.17	.33	.45	.53	.51	.49
.18	.25	.44	.63	.76	.67	.64
.22	.24	.34	.68		.87	.80
.17	.25	.22	.37	.58	.56	.42
.20	.20	.13	.15	.18	.25	.25

5.6

.22	.25	.34	.39	.37	.32	.27
.28	.31	.38	.45	.52	.53	.45
.26	.27	.48	.64	.73	.72	.69
.21	.34	.59	.70	.75	.83	.84
.15	.18	.29	.55	.87		.93
.13	.19	.18	.33	.58	.58	.37
.16	.16	.09	.10	.14	.17	.14

5.7

.22	.26	.32	.36	.34	.29	.22
.28	.30	.35	.41	.48	.50	.42
.27	.27	.45	.58	.66	.68	.66
.24	.34	.53	.59	.65	.75	.79
.30	.32	.43	.66	.80	.93	
.28	.34	.34	.51	.75	.77	.57
.26	.29	.22	.26	.31	.33	.28

6.1

.36	.30	.27	.24	.19	.12	.08
.38	.33	.32	.23	.16	.12	.09
.39	.33	.23	.16	.18	.22	.20
.51	.36	.14	.12	.24	.21	.23
.90	.85	.67	.41	.17	.13	.28
	.92	.81	.66	.53	.54	.64
.86	.90	.88	.82	.79	.77	.72

6.2

.25	.20	.17	.16	.13	.07	.03
.26	.23	.24	.17	.12	.10	.06
.27	.27	.20	.17	.21	.24	.22
.44	.35	.17	.18	.29	.25	.25
.91	.93	.79	.51	.25	.19	.34
.92		.91	.78	.63	.62	.73
.81	.89	.92	.90	.87	.85	.81

6.3

.12	.09	.08	.08	.07	.04	−.03
.14	.11	.15	.10	.06	.05	.02
.15	.18	.15	.14	.16	.19	.17
.32	.28	.16	.19	.24	.21	.22
.84	.94	.92	.58	.22	.18	.34
.81	.91		.90	.71	.69	.81
.74	.78	.86	.92	.92	.90	.85

6.4

.00	−.01	−.01	−.01	−.01	−.03	−.08
.03	.01	.05	.03	.04	.04	.00
.04	.06	.10	.15	.18	.20	.18
.18	.17	.17	.24	.28	.26	.27
.70	.81	.94	.79	.37	.33	.51
.66	.78	.90		.90	.87	.92
.59	.62	.70	.81	.85	.83	.78

6.5

.07	.10	.12	.13	.11	.07	.02
.10	.12	.16	.16	.19	.20	.15
.12	.14	.24	.31	.35	.36	.34
.19	.23	.32	.36	.42	.43	.45
.58	.65	.80	.87	.58	.58	.75
.53	.63	.71	.90		.97	.89
.48	.49	.54	.62	.68	.67	.61

6.6

.12	.16	.20	.20	.17	.13	.07
.16	.19	.22	.21	.25	.27	.21
.18	.20	.28	.35	.38	.41	.39
.25	.27	.34	.37	.43	.46	.50
.59	.64	.77	.83	.56	.58	.77
.54	.62	.69	.87	.97		.92
.48	.50	.54	.63	.69	.69	.62

6.7

.10	.11	.13	.13	.11	.08	.02
.09	.11	.14	.10	.11	.13	.10
.12	.13	.15	.19	.22	.26	.26
.22	.20	.19	.26	.32	.30	.34
.67	.74	.84	.79	.42	.37	.57
.64	.73	.81	.92	.89	.92	
.54	.60	.66	.77	.83	.85	.77

7.1

.31	.24	.23	.20	.17	.12	.04
.32	.26	.29	.20	.12	.11	.07
.32	.26	.21	.13	.16	.19	.17
.41	.31	.14	.15	.23	.20	.21
.80	.75	.61	.39	.20	.16	.26
.86	.81	.74	.59	.48	.48	.54
	.88	.84	.76	.73	.70	.67

7.2

.32	.23	.20	.18	.14	.09	.02
.30	.24	.25	.18	.12	.10	.07
.30	.25	.18	.11	.16	.20	.19
.42	.30	.11	.12	.26	.22	.24
.83	.80	.63	.40	.20	.16	.29
.90	.89	.78	.62	.49	.50	.60
.88		.90	.82	.79	.76	.74

7.3

.20	.14	.12	.12	.11	.08	.01
.19	.17	.20	.15	.10	.08	.06
.24	.24	.17	.10	.14	.19	.18
.38	.30	.12	.09	.18	.16	.18
.84	.84	.71	.41	.13	.09	.22
.88	.92	.86	.70	.54	.54	.66
.84	.90		.91	.88	.83	.81

7.4

.14	.10	.12	.12	.11	.09	.02
.12	.15	.20	.16	.13	.11	.10
.20	.25	.21	.16	.17	.20	.21
.36	.32	.19	.14	.19	.17	.19
.82	.88	.82	.49	.15	.10	.26
.82	.90	.92	.81	.62	.63	.77
.76	.82	.91		.96	.92	.87

7.5

.15	.11	.12	.12	.11	.10	.04
.13	.15	.18	.15	.13	.12	.11
.19	.23	.19	.16	.18	.22	.24
.35	.31	.18	.16	.22	.20	.23
.79	.86	.84	.54	.18	.14	.31
.79	.87	.92	.85	.68	.69	.83
.73	.79	.88	.96		.95	.89

7.6

.14	.10	.11	.11	.10	.07	.01
.09	.10	.15	.10	.07	.07	.06
.13	.16	.13	.12	.13	.17	.17
.27	.24	.12	.18	.25	.19	.20
.76	.84	.83	.57	.25	.17	.33
.77	.85	.90	.83	.67	.69	.85
.70	.76	.83	.92	.95		.92

7.7

.10	.05	.07	.08	.08	.08	.05
.04	.06	.10	.05	.03	.03	.06
.07	.14	.07	.08	.12	.15	.15
.22	.21	.07	.19	.26	.17	.19
.70	.81	.78	.55	.25	.14	.28
.72	.81	.85	.78	.61	.62	.77
.67	.74	.81	.87	.89	.92	
