# Supplementary material for: Multivariate Longitudinal Modeling of Macular Ganglion Cell Complex: Spatiotemporal Correlations and Patterns of Longitudinal Change
Source: Ophthalmol Sci. 2022 Jun 16;2(3):100187. doi: 10.1016/j.xops.2022.100187 (PMC9559093; doi:10.1016/j.xops.2022.100187)
Supplement: Supplemental Fig S2B [file mmc4.pdf]

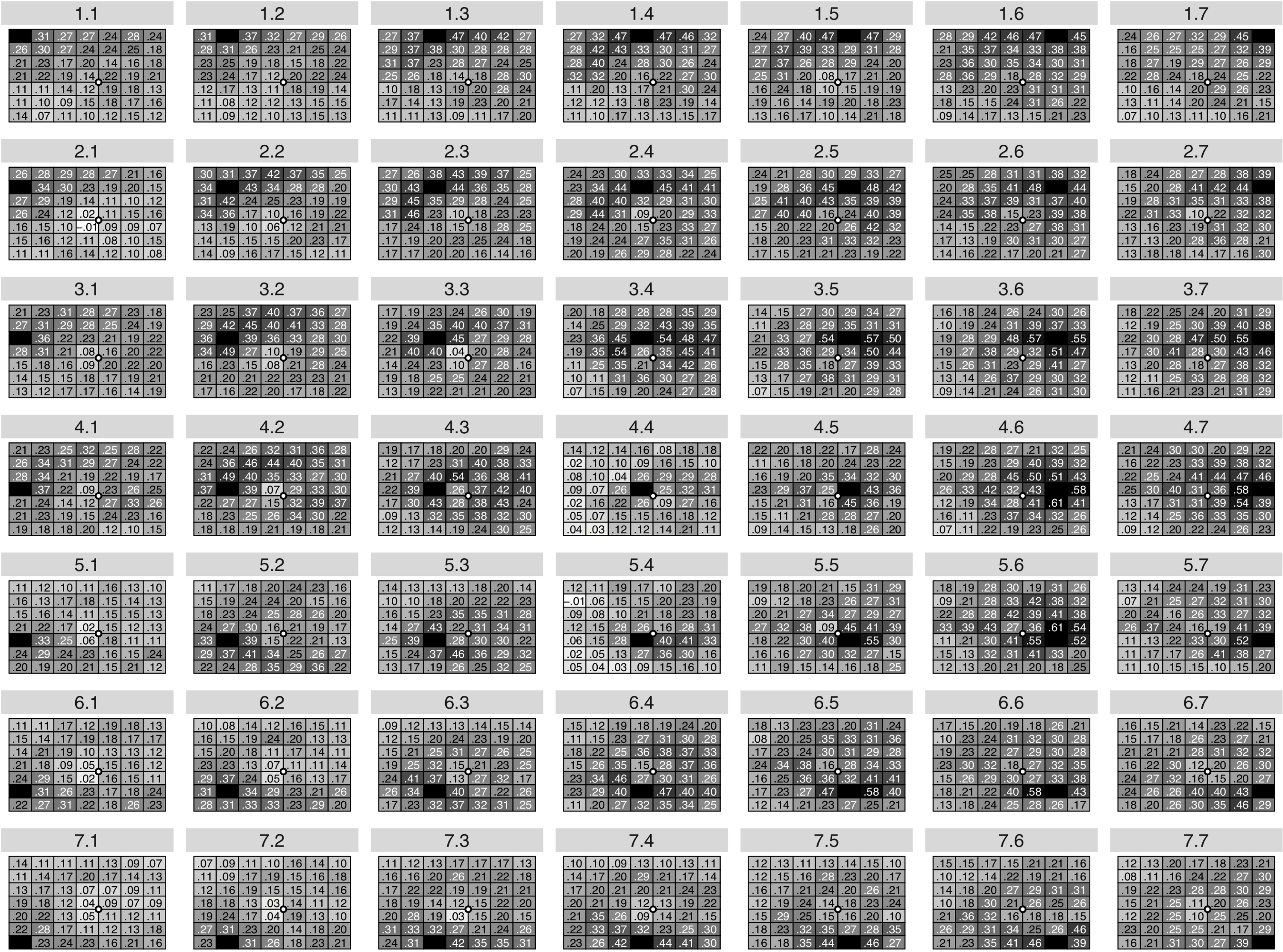
1.1
1.2
1.3
1.4
1.5
1.6
1.7
2.1
2.2
2.3
2.4
2.5
2.6
2.7
3.1
3.2
3.3
3.4
3.5
3.6
3.7
4.1
4.2
4.3
4.4
4.5
4.6
4.7
5.1
5.2
5.3
5.4
5.5
5.6
5.7
6.1
6.2
6.3
6.4
6.5
6.6
6.7
7.1
7.2
7.3
7.4
7.5
7.6
7.7
