# Supplementary material for: Multivariate Longitudinal Modeling of Macular Ganglion Cell Complex: Spatiotemporal Correlations and Patterns of Longitudinal Change
Source: Ophthalmol Sci. 2022 Jun 16;2(3):100187. doi: 10.1016/j.xops.2022.100187 (PMC9559093; doi:10.1016/j.xops.2022.100187)
Supplement: Supplemental Table S1 [file mmc10.pdf]

eTable 1. Summaries of the posteriors for the transformed parameters for our model.

Parameter	Symbol	Mean	SD	2.5%	97.5%
**Mean of Parameter**					
Population Intercept	μ_1	73.05	1.91	69.31	76.77
Log SD of Random Intercepts	$\frac{1}{2}\mu_2$	2.631	0.052	2.529	2.734
Population Slope	μ_3	-0.357	0.041	-0.438	-0.277
Log SD of Random Slopes Adjusted for Random Intercepts	$\frac{1}{2}\mu_4$	-0.300	0.056	-0.409	-0.190
Regression Coefficient for Random Slope Given the Random Intercept	μ_5	-0.0155	0.0020	-0.0194	-0.0117
Log Mean of Random Residual SD	μ_6	0.658	0.019	0.620	0.697
Log SD of Random Residual SD	μ_7	-0.327	0.041	-0.411	-0.248
**SD of Parameter**					
Population Intercept	$\sqrt{\Sigma_{11}}$	13.29	1.37	10.93	16.22
Log SD of Random Intercepts	$\frac{1}{2}\sqrt{\Sigma_{22}}$	0.358	0.038	0.292	0.440
Population Slope	σ_3	0.266	0.030	0.214	0.332
Log SD of Random Slopes Adjusted for Random Intercepts	$\frac{1}{2}\sqrt{\Sigma_{33}}$	0.362	0.042	0.289	0.452
Regression Coefficient for Random Slope Given the Random Intercept	σ_5	0.0119	0.0014	0.0095	0.0149
Log Mean of Random Residual SD	σ_6	0.127	0.014	0.102	0.157
Log SD of Random Residual SD	σ_7	0.227	0.035	0.165	0.303
**Correlations**					
Population Intercept and Log Variance of Random Intercepts	$\frac{\Sigma_{12}}{\sqrt{\Sigma_{11}\Sigma_{22}}}$	0.839	0.045	0.736	0.910
Population Intercept and Log Variance of Random Slopes Adjusted for Random Intercepts	$\frac{\Sigma_{13}}{\sqrt{\Sigma_{11}\Sigma_{33}}}$	0.756	0.066	0.606	0.863
Log Variance of Random Intercepts and Log Variance of Random Slopes Adjusted for Random Intercepts	$\frac{\Sigma_{23}}{\sqrt{\Sigma_{22}\Sigma_{33}}}$	0.835	0.051	0.718	0.914

SD = standard deviation
